# Hybrid VAE-Transformer-based multi-task learning for cyberattack detection and fault classification in electric vehicle charging stations

**DOI:** 10.3389/frai.2026.1934106

**Published:** 2026-10-07

**Authors:** R. Vinitha, R. Senthil Kumar

**Affiliations:** School of Electrical Engineering, Vellore Institute of Technology, Chennai, Tamil Nadu, India

**Keywords:** cyberattack, deep learning, electric vehicle charging station, multi-task learning, physical fault classification

## Abstract

Electric vehicle (EV) charging stations have become a complex cyber-physical system, where the sensor network is susceptible to both hardware failures and cyberattacks. Both produce similar data patterns, making them difficult to distinguish. The majority of the approaches have addressed only cyberattacks or faults and rely on recurrent architectures, which are hard to capture long-term temporal patterns. In this study, a multi-task learning framework for cyberattack detection and fault classification is proposed based on four hybrid deep learning models, namely Variational Autoencoder–Long Short-Term Memory (VAE-LSTM), VAE–Gated Recurrent Unit (VAE-GRU), VAE–Generative Adversarial Network (VAE-GAN), and VAE-Transformer. The framework is tested on a publicly available EV Internet of Things (IoT) predictive-maintenance dataset with six threat scenarios like Fast Gradient Sign Method (FGSM), Projected Gradient Descent (PGD), Replay attacks, Man-in-the-Middle (MitM) attacks, Message Manipulation attacks, and a Flatline sensor fault. The results show that the VAE-Transformer achieved an Area Under the Curve–Receiver Operating Characteristic (AUC-ROC) of 99.76%, a binary F1-score of 99.03%, an attack-type Macro-F1 of 97.29%, an FPR@95% of 0.41%, and a Matthews correlation coefficient (MCC) of 98.20%. Furthermore, the low values of orthogonality and Mutual Information (MI) loss demonstrate that the proposed hybrid deep learning algorithm successfully separates cyberattack and physical fault features, thereby improving overall detection performance.

## 1 Introduction

The transportation sector accounts for almost 24% of global CO_2_ emissions, and the rapid worldwide adoption of electric vehicles (EVs) is an important pathway for reducing transportation-related emissions (Gorthi and Jyothi, 2026). According to the International Energy Agency (IEA) Global EV Outlook 2026, the global market for EVs sold in 2025 was already more than 20 million, which is around 25% of the world's new car sales. By 2035, it is estimated that EVs will number over 450 million, and electricity consumption for EV charging will increase significantly during this timeframe [International Energy Agency (IEA), 2026]. The public charging infrastructure is also improving, as EV Charging Stations (EVCSs) are becoming an important part of today's electricity and smart grid infrastructure, BloombergNEF reports (BloombergNEF, 2026). With the growth in EV usage and charging demand, the resilience and security of EVCSs are critical.

Today's EVCSs are cyber-physical systems that combine power electronic converters, embedded sensors, communication networks, and control systems, communicating with each other using protocols such as Open Charge Point Protocol (OCPP) and International Organization for Standardization (ISO) 15118 (Gorthi and Jyothi, 2026). They facilitate real-time communication among EVs, charging stations, service providers, and the smart grid for applications such as dynamic pricing, remote diagnostics, demand response, and Vehicle-to-Grid (V2G) operation. However, the benefits of enhanced connectivity come at the cost of a higher attack surface for EVCSs. Cyberattacks may alter sensor measurements, messages transmitted over communications links, or control instructions for equipment, leading to incorrect charging actions, equipment damage, and unintended impacts on grid operations (Waqar et al., 2026). Hence, it is important to monitor the cyber-physical status of EVCSs to ensure safe and uninterrupted charging operation.

The anomaly detection systems for EV systems included rule-based systems and statistical models such as Autoregressive Integrated Moving Average (ARIMA) and Principal Component Analysis (PCA), where the assumptions of linear relationships and stationary distributions are often not met in real-time EV environments (Wang et al., 2024). All these methods generate a high proportion of false positives and cannot change their operating conditions. Classical machine learning techniques like Support Vector Machine (SVM), K-Nearest Neighbor (KNN), and Random Forests (RFs) achieved good generalization results. Such approaches heavily depend on the manually engineered features and require a great deal of domain knowledge. They may fail to recognize fine-grained patterns or extract salient features when operating conditions fluctuate. In the end, these models usually address a specific objective, such as identifying a cyberattack or a physical failure, but not both. This limitation can affect their performance in practical EVCS installations where cyberattacks and physical failures can happen simultaneously.

Monitoring in EVCSs requires joint monitoring of the cyberphysical aspects, as EV charging can impact the distribution network voltage, Transformer loading, and power quality. Temporal deep learning models, such as attention-based architectures (Balakumar et al., 2024; Chadha et al., 2026; Jiang et al., 2025; Ghosh and Roy, 2024) and Bidirectional Long Short-Term Memory (Bi-LSTM) have thus been integrated into forecasting and energy-management approaches. Thus far, the majority of these systems assume that the data from the sensors for control and decision-making is reliable. Cyberattacks can invalidate this assumption by altering measurements used by energy-management and control systems (Ghosh and Roy, 2024). Therefore, it is important that a reliable EVCS monitoring system not only identifies any abnormal sensor behavior but also determines whether the abnormal behavior is caused by a cyberattack or a physical fault.

This is difficult to distinguish since similar patterns can be seen in sensor time-series measurements as a result of cyberattacks and physical defects (Rahman et al., 2024). The multi-agent Deep Reinforcement Learning (DRL) and Koopman-based approaches have been studied recently for detection and attack isolation of cyberattacks (Ramul et al., 2025; Xia et al., 2026). In addition, methods in the field of Explainable Artificial Intelligence (XAI) can be used to aid in the interpretation of attack-related decisions, such as SHapley Additive exPlanations (SHAP) and Local Interpretable Model-Agnostic Explanationsb (LIME), which described (Afrin et al., 2025). Current solutions, however, mainly address specific threat classes or single-system goals and seldom offer a holistic approach that can detect cyberattacks and simultaneously categorize a physical fault.

In contrast to Feature engineering, Deep Learning (DL) eliminates the need for it by learning representations directly from raw sensor data relevant to the task. Convolutional Neural Networks (CNNs) effectively capture short-term fault characteristics and local spatial patterns, improving fault detection performance. Sequential temporal dependencies across charging sessions can be captured by LSTM and Gated Recurrent Unit (GRU) networks and have been extensively used in EV fault detection and energy management (Pawlik et al., 2025). For V2G fault detection, hybrid CNN-LSTM architectures are used to leverage both approaches, whereas attention-based variants selectively weight informative time steps to estimate the battery State-of-Health (SoH) and model the battery degradation process (Peng et al., 2026; Saranathan et al., 2026). Ensemble learning frameworks estimate SoH and Remaining Useful Life (RUL) simultaneously, enhancing the robustness of SoH estimation for different battery chemistries. Inverter fault detection and EV drivetrain predictive maintenance are also extended to machine learning-based frameworks (Xu et al., 2025; Palaiologou et al., 2025). There is a fundamental constraint in the structure of LSTM and GRU networks: information travels sequentially through the hidden states, representing past context that is progressively diluted. Recurrent models are susceptible to low-rate data injection attacks that cause incorrect sensor data to be recorded over hours, and the detected anomaly may be some time removed from the causal signal. Daah et al. (2026) proposed that the CNN-BiLSTM-Attention architecture partially addresses this issue by combining CNN feature extraction with a bidirectional LSTM structure, which has demonstrated competitive performance in smart grid intrusion detection. These architectures are based on recurrent networks and are limited in their ability to capture long-term temporal information and achieve high inference speed. However, for recurrent architectures, information is presented sequentially, which limits their capacity to learn long-term temporal dependencies and relationships efficiently. Furthermore, traditional Variational Autoencoder (VAE) models yield a latent representation that is treated as a probability distribution, but typically do not explicitly distinguish between cyber-related and physical-fault-based attributes. Additionally, current EV security models remain limited in uncertainty-aware representation, interpretability, and their ability to simultaneously handle diverse cyber-physical scenarios (Ghoreishi et al., 2026; Terruggia et al., 2025).

The majority of the existing EVCS research focuses on detecting a cyberattack or on diagnosing a physical fault and applying them to separate scenarios, and it is difficult to distinguish between the cyber anomaly and the physical fault when the sensor pattern is similar. Traditional methods are also limited in their ability to isolate cyber-physical characteristics and capture long-range temporal dependencies. This encourages the development of a common VAE-Transformer model that learns disentangled cyber-physical representations and temporal dependencies, while simultaneously detecting cyberattacks and classifying physical faults. Table 1 summarizes the existing approaches, which are generally targeted at individual aspects of EV system security or fault diagnosis. Few studies unify probabilistic representation learning, cyber-physical disentanglement, long-range temporal modeling, and multi-task classification in a single framework. This gap leads to the development of the proposed VAE-Transformer framework for simultaneous cyberattack detection and physical-fault classification.

**Table 1:** Comparison of existing methods and VAE–Transformer framework.

References	Method	Fault detection	Cyber detection	Long-range temperature	Multi-task	Probabil-istic	Disen-tangled	Interpret-able	Scalability	Limitations	Research gap
Gorthi and Jyothi (2026)	CNN-LSTM	✓	✗	✗	✗	✗	✗	✗	Partial	Energy-management/fault only, no cyberattack detection	Lacks unified security–fault modeling, no adversarial-attack resilience
Balakumar et al. (2024)	Bi-LSTM	✗	✗	✗	✗	✗	✗	✗	Partial	Single-task, no attack detection, weak on long sequences	No anomaly/attack detection, not designed for fault–cyber fusion
Jiang et al. (2025)	Koopman	✗	✓	✗	✗	✗	✗	✓	High	Cyber only, residual-based, no fault classification	No probabilistic latent space, cannot separate cyber vs. physical-fault causes
Ghosh and Roy (2024)	XAI Detect.	✗	✓	✗	✗	✗	✗	✓	Low	Cyber only, no fault classification, not probabilistic	Interpretable yet single-domain, no disentangled or multi-task detection
Rahman et al. (2024)	Multiagent DRL	✗	✓	✓	✗	✗	✗	✗	Partial	Complex multi-agent training, cyber only, no fault classification	No fault attribution, heavy training, lacks generative latent representation
Pawlik et al. (2025)	Attn-SOH	✓	✗	✓	✗	✗	✗	✗	Low	Health estimation only, no cyberattack detection, and single-task	Security-agnostic, no cyberattack or multi-task capability
Peng et al. (2026)	Ensemble	✓	✗	✗	✗	✗	✗	✗	Low	Fault only, no temporal modeling, poor scalability	No temporal/attention modeling, not extendable to cyber detection
Palaiologou et al. (2025)	Zero-Trust AI	✗	✓	✗	✗	✗	✗	✗	Low	Access-control focused, no fault detection, low scalability	Operates at access layer, no signal-level fault or anomaly detection
Mitikiri et al. (2025)	CNN-LSTM Fusion	✓	✗	✗	✗	✗	✗	✗	Partial	Multi-fault diagnosis only, no cyber detection, no long-range attention	No cyber detection, long-range attention, or probabilistic outputs
Proposed study	VAE-Transformer	✓	✓	✓	✓	✓	✓	✓	High	Higher training complexity, validated on a single dataset	High training complexity and no cross-dataset validation

The motivation for integrating the VAE-Transformer architectures stems from the nature of cyber-physical systems in EV charging stations. Both a cyberattack and a physical fault may create similar patterns in sensor measurements, making it difficult to distinguish between them reliably. The VAE is used to train a small, disentangled latent representation to help separate attack- and fault-related features. In the meantime, the Transformer is used to learn the long-term temporal dependence among multivariate sensor sequences. Hence, the proposed VAE-Transformer architecture specifically caters to the combined demands of feature disentanglement and temporal modeling at greater distances for cyberattack detection and fault classification of EVs.

The main objective of this study is to develop a hybrid VAE-Transformer-based multi-task learning framework for simultaneous cyberattack detection and fault classification in EV charging stations. Figure 1 shows the overall architecture of the proposed VAE-based hybrid deep learning framework. The framework begins by collecting data from EVs via their sensors, then preprocesses it, and finally extracts informative latent features using a VAE. The hybrid deep learning model (LSTM, GRU, Generative Adversarial Network [GAN], and Transformer) is used to analyze these features and capture discriminative feature representations. Finally, the framework is capable of not only detecting cyberattacks [such as Fast Gradient Sign Method (FGSM), Projected Gradient Descent (PGD), Replay, Man-in-the-Middle (MitM), Message manipulation attack] but also categorizing physical faults (Flatline). The VAE-Transformer is likely to be the most effective model due to its built-in self-attention mechanism, which can learn long-range temporal dependencies while leveraging parallel computing and introducing feature separation.

**Figure 1:**
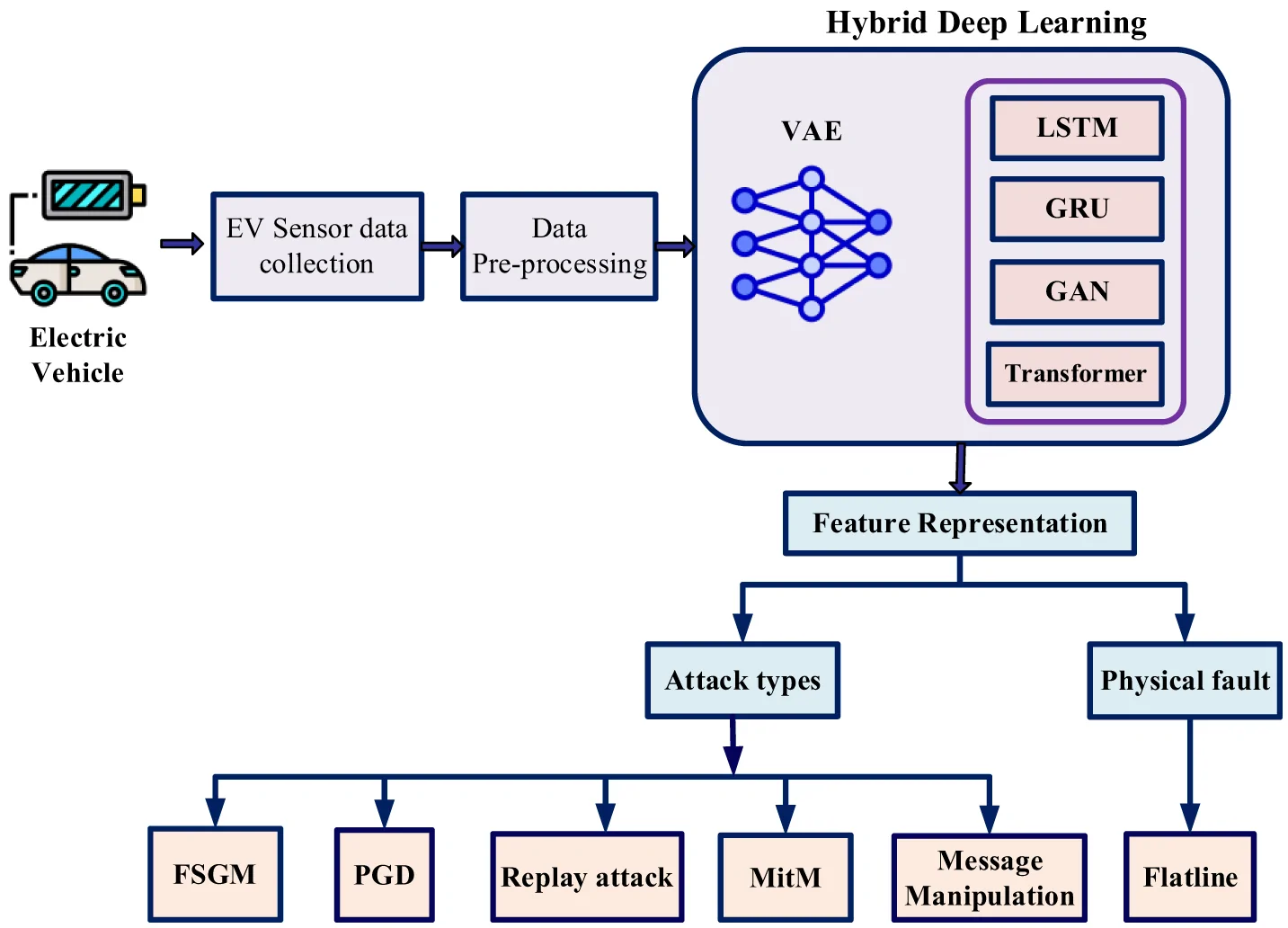
Proposed VAE-based hybrid deep learning framework.

The main contributions of this study are as follows:

- A hybrid deep learning framework is proposed by integrating a Variational Autoencoder (VAE) with various DL architectures, including LSTM, GRU, GAN, and Transformer.
- A disentangled latent space representation is learned that separates cyber-related and physical fault-related features, reducing misclassification between cyberattacks and physical faults.
- A probabilistic VAE-based feature learning strategy is employed to enhance feature representation, improve detection accuracy, and reduce uncertainty in the learned latent features.
- The Transformer self-attention mechanism is incorporated to capture long-range temporal dependencies in sensor data, resulting in superior cyberattack detection and fault classification compared with DL models.
- A domain-aware consistency mechanism is incorporated to preserve the relationships among physically related EV charging sensor variables. This improves the alignment of learned features with the underlying cyber-physical characteristics of EV charging infrastructure.

This study is organized as follows: Section 2 describes the Cyberattack and physical fault scenarios. The hybrid VAE-based deep learning frameworks are presented in Section 3. The discussion on the performance evaluation metrics of the proposed system is in Section 4. The obtained results are presented in Section 5. Section 6 compares the proposed study with existing studies. The conclusion and the future research directions are outlined in Section 7.

## 2 Types of attacks and faults in EV charging networks

This section describes six different threat scenarios: Five cyberattacks (FGSM, PGD, Replay, MitM, and Message Manipulation attacks) and one physical fault (Flatline). All cyberattack scenarios are synthetically injected into clean EV sensor recordings, which are gathered from IoT-enabled EVs in various settings (EV Data Center, 2025). Each injection is designed to remain within a physically realistic sensor range, ensuring that the resulting attack samples cannot be easily identified through simple sensor-range checks.

### 2.1 Fast Gradient Sign Method (FGSM)

FGSM is an adversarial attack that adds a small, mathematically crafted modification to the original sensor readings to mislead the target detection model. The key idea is that the disturbance is structured rather than random noise, but it is calculated in a direction that most likely causes a mistake to the target detection model (Liu et al., 2026). In a formal sense, if the input window is supplied with a loss function (*J*) then the adversarial sample is created.

The adversarial input(*x*_*adv*_)can be expressed in Equation 1.


$$\begin{array}{l} x_{adv}=\;x+\varepsilon \;.\;sign\left(\nabla_{x}J\left(x,y\right)\right) \end{array}$$ (1)

where, *x* is the original sensor data, ε controls the size of the perturbation, *J*(*x, y*) is the loss function, (∇_*x*_*J*(*x, y*)) is the gradient of the loss with respect to the input, and sign(.) determines the direction of the perturbation added to the input.

The FGSM-corrupted data mimic the output of real sensors almost completely from a detection perspective. The changes are not large enough to trigger any threshold-based decision, but are targeted at the model's decision boundary. Attacks done through FGSM take only one step and therefore are easy to quickly deploy at scale.

### 2.2 Projected Gradient Descent (PGD)

The PGD iteratively updates the perturbation to create a stronger adversarial attack without exceeding the permitted perturbation limit (Lyu et al., 2024). The first step of PGD is from a clean input (*x*). The adversarial sample after the next iteration $\left(x_{adv}^{k+1}\right)$ is given in Equation 2.


$$\begin{array}{l} x_{adv}^{k+1}=\Pi_{x}\left(x_{adv}^{k}+\alpha .sign\left(\nabla_{x}J\right)\right) \end{array}$$ (2)

where $x_{adv}^{k}$ is the adversarial sample at the current iteration, α is the step size, *J* is the loss function, ∇_*x*_*J* is the gradient of the loss with respect to the input, and *P*_*x*_(.) projects the perturbed sample back into the allowable perturbation region around the original input.

The adversarial signal generated by PGD is more sophisticated than that generated by FGSM, since PGD iteratively optimizes the perturbation within the desired range, enabling the creation of more effective adversarial examples. As for the corrupted sensor readings, they are statistically very close to normal operating data, which makes PGD the most difficult attack to detect. Nearly all the confusion in the normal-class samples shown in the results is due to samples attacked by PGD; this stems from PGD's ability to closely mimic normal sensor behavior, making them inherently difficult to distinguish.

### 2.3 Replay—Re-injected legitimate sensor data

A Replay attack is the easiest of all attacks, but relies on a secret and powerful weakness, and real data from the past is not the same as real data from now. The attacker does not create any sensor values but instead obtains legitimate sensor readings from a previous operating session and replays them into the current data stream at the wrong time (Liu et al., 2011).

Formally, a segment of sensor data from time *t–d* is substituted for the live readings in the window [*t*_*start*_, *t*_*start*_ + L_*r*_]: The corresponding expression is given in Equation 3.


$$\begin{array}{l} x_{t}=\;x_{t-d}\text{ for t }\varepsilon \left[t_{start},t_{start}+L_{r}\right] \end{array}$$ (3)

where *x*_*t*_ is the sensor reading at the current time *t*, *x*_*t*−*d*_ is the previously recorded sensor reading delayed by d time steps; d is the replay delay, *t*_*start*_ is the time at which the replay attack begins, *L*_*r*_ is the duration of the replay attack, and t ε [*t*_*start*_, *t*_*start*_ +*L*_*r*_] indicates that the replayed data are used throughout the attack interval.

This temporal characteristic of the Replay attack provides a compelling case for long-range self-attention. The reason is that a recurrent model with local windows of recent readings will not notice an unusual situation, whereas a model that compares the current segment against the wider temporal context of the session will notice an unusual situation. Only a model that considers both the current data and earlier sensor readings can detect that the readings are outdated.

### 2.4 Man-in-the-Middle (MitM) attack

A Man-in-the-Middle (MitM) attack occurs when an attacker covertly intercepts communication between the EV, charging station, and backend system and can eavesdrop on or alter the information exchanged. In the IoT-enabled EV charging environment, an attacker can eavesdrop on the charging commands, sensor measurements, authentication messages, or energy-related information exchanged between the communication endpoints. This may result in false charging decisions, unauthorized control, or interruption of normal charging operation.

This framework introduces a MitM attack by synthetically modifying communication-related sensor data while keeping the values within the real operating range. The attack aims to maintain the general characteristics of authentic sensor signals while introducing inconsistency in the transmitted information. This makes the attack difficult to detect by simple threshold-based detection methods.

The MitM attack can be represented in Equation 4.


$$\begin{array}{l} x_{t}^{MitM}=x_{t}+\delta_{t} \end{array}$$ (4)

where *x*_*t*_ represents the original sensor/communication data at time t, δ_*t*_ represents the communication-level modification introduced by the attacker, and $x_{t}^{MitM}$ denotes the resulting compromised data. The inclusion of MitM provides an additional evaluation of the model's ability to detect communication-level cybersecurity threats in EV charging networks.

### 2.5 Message Manipulation attack

A Message Manipulation (MsgManip) attack is an attack in which an attacker modifies legitimate messages exchanged between EV charging components, without necessarily disrupting the communication channel. The manipulated messages in an EV charging network can be charging status, power commands, voltage/current information, authentication data, or control signals. Small changes can lead to incorrect system decisions and compromise the safety and reliability of the charging process.

In this study, the Message Manipulation attack is synthetically generated by introducing controlled modifications into legitimate sensor or communication messages. The modified values are constrained to remain within realistic ranges so that the attack resembles a plausible communication disturbance rather than an obviously abnormal signal. The manipulated data are then assigned to the corresponding attack class and evaluated by the proposed deep learning framework.

The manipulated message can be expressed in Equation 5.


$$\begin{array}{l} x_{t}^{Msg}=x_{t}+\Delta_{t} \end{array}$$ (5)

where *x*_*t*_ is the original legitimate message, Δ_*t*_ represents the injected manipulation, and $x_{t}^{Msg}$ is the resulting compromised message. This attack scenario evaluates the proposed model's ability to distinguish legitimate EV charging communications from subtly modified messages.

### 2.6 Physical fault: flatline

Inspecting all errors in EV charging stations is not necessary to carry out an attack. Physical components fail sensors stopping, transducers getting stuck, communication modules freezing. A flatline fault mimics a hardware failure: a channel, or a set of channels from a particular sensor, operates at a fixed value over an extended period of time, setting that value without ever continuing to update the sensor readings (Mahapatro and andKhilar, 2013).

The corresponding equation is given in Equation 6.


$$\begin{array}{l} x_{t,d}=\;c_{d}\text{ for t }\varepsilon \left[t_{start},t_{start}+L_{f}\right] \end{array}$$ (6)

where *x*_*t, d*_ is the reading of sensor d at time t, *c*_*d*_ is the constant value assigned to the faulty sensor, *t*_*start*_ is the time at which the flatline fault begins, *L*_*f*_ is the duration of the fault, and t ε [*t*_*start*_, *t*_*start*_ + *L*_*f*_] indicates that the sensor output remains fixed throughout the fault interval. The Flatline fault has been added due to its resemblance to some cyberattacks, which makes it difficult to differentiate between the two.

The majority of cyberattacks aim to disrupt sensors by including only small or realistic changes to sensor data. The minor perturbations introduced by FGSM and PGD, replay attacks using previously recorded valid sensor data, and flat-line attacks with patterns that can resemble data suppression attacks are all small perturbations. The small perturbations are introduced by FGSM, PGD, replay attacks using previously recorded valid sensor data, and flat-line attacks using patterns that can look likeusing previously recorded valid sensor data, and flat-line attacks using patterns that can resemble data suppression attacks. Therefore, both long-range temporal dependencies and probabilistic feature representations need to be learned to distinguish cyberattacks from physical faults. To address this challenge, the VAE-Transformer framework is designed to learn disentangled probabilistic latent representations of cyber-physical features with the VAE, and to model long-range temporal dependencies by applying a self-attention mechanism to accurately detect and classify the attacks.

## 3 Hybrid deep learning architectures

All four DL models are trained with the same training-test split, batch composition, optimizer, and the number of epochs. All generate a binary cyberattack classification (normal vs. attack) and a multi-class fault classification. The differences between all four proposed models and the single-task approaches lie in their multi-task learning framework.

### 3.1 VAE-based feature learning framework

It is important to describe the common architectures before discussing the differences between the four proposed models. The common basis is the Variational Autoencoder (VAE), which serves as the common encoder-decoder architecture.

The encoder compresses the raw EV sensor data into a low-dimensional latent representation, while the decoder reconstructs the original input from the learned latent variables (Rengarajan, 2025). Unlike a conventional autoencoder, which maps each input to a deterministic latent vector, the VAE learns a probabilistic latent representation by modeling the latent variables as a Gaussian distribution characterized by a mean and variance. This probabilistic formulation effectively captures the inherent uncertainty in EV sensor data and enables the learning of more robust and generalized latent features for subsequent cyberattack detection and fault classification.

The VAE is trained by minimizing the loss with the latent distribution, encoder, and loss function expressed in Equations 7–9.


$$\begin{array}{l} \text{Latent distribution}:Z\sim N\left(\mu ,\text{diag}\left(\sigma^{2}\right)\right) \end{array}$$ (7)

$$\begin{array}{l} \text{Encoder}:\;\left[\mu ,\log\sigma^{2}\right]=f_{\theta }\left(x\right) \end{array}$$ (8)

$$\begin{array}{l} \text{Loss function}:L_{VAE}=E_{q\left(z|x\right)}\left[{{||x-\hat{x}||}^{2}}_{2}\right]+D_{KL}\left(q\left(z|x\right)||p\left(z\right)\right) \end{array}$$ (9)

The first term represents the reconstruction error, which is the quality of the VAE's approximation of the input sensor window when reconstructing it from the compressed output. A high reconstruction error indicates that the input deviates from the normal data distribution learned by the VAE, suggesting that it may correspond to an anomalous event such as a cyberattack or sensor fault (Mitikiri et al., 2025). The second term is the KL-divergence, which regularizes the latent space to be close to a standard Gaussian distribution. This will help keep the model from memorizing the training examples and encourage it to learn more general representations of normal operation. Figure 2 shows the architecture of the Variational Autoencoder (VAE), in which the input is encoded into a probabilistic latent representation parameterized by the mean and standard deviation. The latent vector is then used to form the input and reconstruction, and KL divergence losses are used to optimize the learned latent representation.

**Figure 2:**
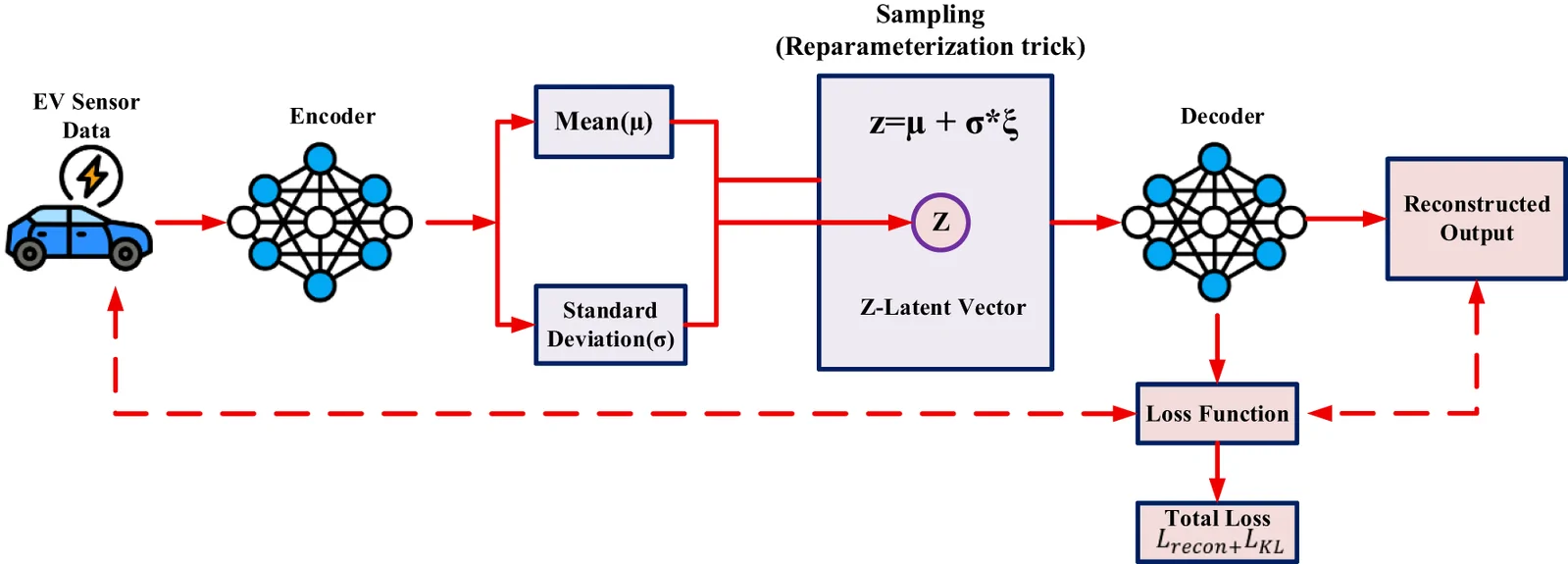
Architecture of the Variational Autoencoder (VAE).

### 3.2 LSTM

The temporal dependency in EV sensor data is modeled as a baseline using an LSTM. It has a gated memory mechanism that allows it to retain relevant historical information, which can make it inefficient at capturing long-range temporal dependencies when processing data sequentially (Chen et al., 2026).

### 3.3 GRU

GRU is a simpler recurrent network that uses update and reset gates to regulate the flow of temporal information, compared with LSTM. It is simpler, which allows faster training without compromising its temporal modeling ability (Balakumar et al., 2026).

### 3.4 GAN

GAN is an adversarial learning framework that involves training a generator and discriminator to learn complex data distributions. In the VAE-GAN baseline, adversarial training is incorporated into the network to enhance feature learning, alongside VAE reconstruction and latent regularization.

### 3.5 Transformer model

Transformer uses a self-attention mechanism to capture long-range dependencies in sequential data. It processes all input data in parallel, enabling faster and more accurate learning than recurrent models such as LSTM and GRU. The VAE-Transformer model splits the latent space into a cyber subspace and a physical subspace, making it easier to reason about cybersecurity threats and physical hardware faults. This disentanglement is unique to the proposed architecture. The VAE-Transformer is a variant of the VAE model in which the recurrent sequence module is replaced with a Transformer encoder based on a multi-head self-attention module. The Transformer reads all the time steps at once and computes the amount of attention to give to each time step in a sequence of time steps.

To do this, each input time step is projected into three vectors—a Query (Q), a Key (K), and a Value (V):

The attention score between any two-time steps is computed from Equations 10–13.


$$\begin{array}{l} Q=XW_{Q} \end{array}$$ (10)

$$\begin{array}{l} K=XW_{K} \end{array}$$ (11)

$$\begin{array}{l} V=XW_{V} \end{array}$$ (12)

$$\begin{array}{l} Attention\left(Q,K\;,V\right)=Softmax\left(\frac{QK^{T}}{\sqrt{d_{k}}}\right)V \end{array}$$ (13)

Here, *X* is the input sequence, *Q*, *K*, and *V* are the query, key, and value matrices obtained using learnable weight matrices, *W*_*Q*_, *W*_*k*_, and *W*_*v*_. The attention function computes the similarity between queries and keys, applies the Softmax function to obtain attention weights, and uses these weights to generate the output from the value matrix V.

The unique power of this, compared with recurrent models, is that the dependency distance between any two time steps is fixed and independent of how far apart they are in the sequence. This capability allows the model to effectively capture long-range temporal patterns without suffering from information bottlenecks or gradient degradation over extended sequences (Waqar et al., 2026; Makaremi, 2025; Kuang et al., 2025). Moreover, attention mechanisms are effective for long-term dependencies in the battery health prediction task (Zhao et al., 2023).

The VAE-Transformer framework is illustrated in Figure 3. The pre-processed EV sensor data is encoded into disentangled cyber and fault latent representations. The Transformer is used to extract temporal features. In contrast, the decoder is used to reconstruct the input and, simultaneously, to perform binary attack detection and multi-class fault classification.

**Figure 3:**
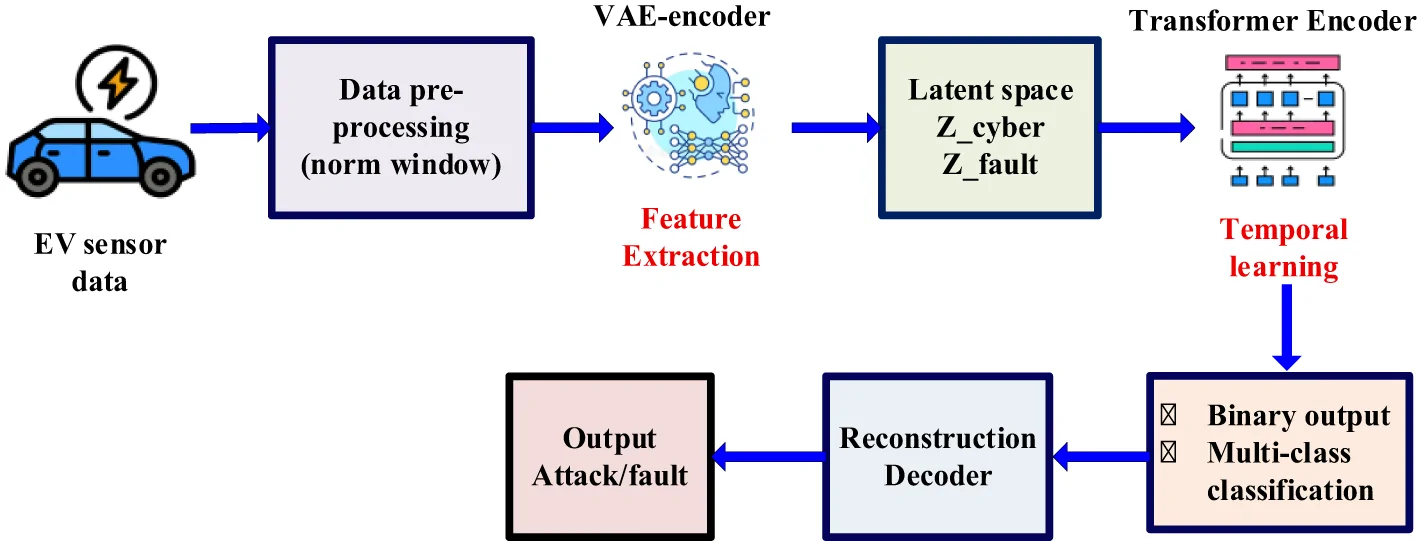
Architecture of the VAE-Transformer framework.

### 3.6 Disentangled latent space

The VAE latent space is explicitly split into two non-overlapping subspaces:

- *Z*_*c*_ (cyber subspace): captures features associated with adversarial manipulations in the sensor stream.
- *Z*_*f*_ (physical subspace): captures features associated with hardware faults and degradation.

This design is inspired by the need to identify cyber-physical anomalies, which has been a challenge in previous EV cybersecurity designs (Xia et al., 2026; Ghosh and Roy, 2025; Sepehrzad et al., 2024). The model is trained to distinguish whether an anomaly is caused by a cyberattack or a physical fault using Mutual Information (MI) minimization and an orthogonality constraint.

### 3.7 EV-domain-aware physical consistency

The proposed framework incorporates EV-domain knowledge by considering the relationships among physically related sensor groups rather than treating all channels independently. Battery variables [state of charge (SoC), State of Health (SoH), voltage, current, and temperature], motor variables [temperature, vibration, torque, Revolutions Per Minute (RPM), and power], and other operating-condition variables exhibit correlated behavior. These relationships are used to improve the distinction between normal operating variations, cyber manipulation, and physical faults.

A domain-consistency loss is expressed in Equation 14:


$$\begin{array}{l} L_{domain}=\frac{1}{G}{\overset{G}{\underset{g=1}{\sum }}||C_{g}\left(x\right)-C_{g}\left(\hat{x}\right)\;||}_{F}^{2} \end{array}$$ (14)

The final training loss function is formulated in Equation 15:


$$\begin{array}{l} L_{total}=L_{Recon}+{\text{λ}}_{KL}\cdot L_{KL}+{\text{λ}}_{BCE}\cdot L_{BCE}+{\text{λ}}_{CE}\cdot L_{CE}\\ \;+{\text{λ}}_{dis}\cdot L_{Dis}+{\text{λ}}_{domain}\cdot L_{domain} \end{array}$$ (15)

This domain-aware constraint enables the model to preserve meaningful relationships between EV sensor variables and strengthens its applicability to EV cyber-physical anomaly detection. The sensor groups used are consistent with the EV telemetry features described in the dataset.

### 3.8 Loss functions

The loss functions facilitate the model to learn accurate features by improving the reconstruction of the input, regularizing the latent space, improving the classification of cyberattack and faults, and separating the cyberattack and physical fault features for better detection performance. First the reconstruction loss (*L*_*RL*_) is given in Equation 16.


$$\begin{array}{l} L_{RL}=\text{E}\left[||\text{x}-\hat{\text{x}}{||}^{2}\right] \end{array}$$ (16)

The reconstruction loss measures the difference between the original input x and the reconstructed output $\hat{x}$. Here, E denotes the expectation and ||x – $\hat{x}||$^2^ represents the squared error. A lower value indicates accurate reconstruction, while a higher value suggests the presence of an anomaly.

The KL Divergence loss (*L*_*KL*_) is expressed in Equation 17.


$$\begin{array}{l} {L_{KL}=D}_{KL}\left(\text{q}\left(\text{z}|\text{x}\right)||\text{p}\left(\text{z}\right)\right) \end{array}$$ (17)

The KL divergence measures the difference between the learned latent distribution q(z|x) and the standard Gaussian prior p(z). It regularizes the latent space, preventing overfitting and improving the model's generalization.

The Binary Cross-Entropy loss (*L*_*BCE*)_ can be expressed in Equation 18.


$$L_{BCE}=-[\text{y}\log\hat{\text{y}}+(1-\text{y})\log(1-\hat{\text{y}})$$ (18)

where y is the true binary label, and ŷ is the predicted probability. The BCE loss penalizes incorrect binary predictions, improving cyberattack and normal-state classification.

The multi-class Cross-Entropy (*L*_*CE*_) is represented in Equation 19.


$$\begin{array}{l} L_{CE}-\text{Σ}{\text{y}}_{\text{k}}\log{\hat{\text{y}}}_{\text{k}} \end{array}$$ (19)

where y_*k*_ is the true class label and ŷ_*k*_ is the predicted probability for class k. This loss penalizes incorrect predictions and improves multi-class fault or attack classification.

The Disentanglement Loss (*L*_*Dis*_) is expressed in Equation 20.


$$\begin{array}{l} {L_{Dis}=\text{ λ}}_{MI}\cdot I\left(Z_{c};Z_{f}\right)+{{\text{λ}}_{orth}\;||Z_{c}^{\wedge }T\;Z_{f}||}_{F}^{2} \end{array}$$ (20)

where *Z*_*c*_ and *Z*_*f*_ represent the cyber-physical latent features, I(.) is Mutual Information, λ_*MI*_ and λ_*orth*_ are weighting factors. The Frobenius norm (F) makes the orthogonality constraint mathematically clearer. This loss separates cyber-physical representations, enabling each latent space to learn only its corresponding information.

The total losses are expressed in Equation 21.


$$\begin{array}{l} L_{total}=L_{RL}+{\text{λ}}_{KL}\cdot L_{KL}+{\text{λ}}_{BCE}\cdot L_{BCE}+{\text{λ}}_{CE}\cdot L_{CE}+{\text{λ}}_{dis}\cdot L_{Dis} \end{array}$$ (21)

The weighting factors λ_*KL*_, λ_*BCE*_, λ_*CE*_, and λ_*dis*_ control the contribution of each loss term and are optimized using the validation set. For the three baseline models, the disentanglement loss is omitted because latent space separation is a unique feature of the proposed architect.

Table 2 shows a comparison of the architectural and training parameters of the four hybrid deep learning models combining a VAE. The sequence learning module and loss function were the only differences between the models, allowing for a fair performance comparison, since they share a common VAE backbone and training settings.

**Table 2:** Architectural and training hyperparameters for all four models.

Parameter	VAE-LSTM	VAE-GRU	VAE-GAN	VAE-transformer
Encoder hidden units	128	128	128	128
Latent dimension (z)	32	32	32	32
Sequence module	LSTM: 2 layers, 128 units	GRU: 2 layers, 128 units	Generator: 2 layers, 128 units Discriminator: 2 layers, 64 units	Transformer: 4 heads, 2 layers, d_model = 128
Activation function	Tanh/Sigmoid gates	Tanh/Sigmoid gates	LeakyReLU (D) ReLU (G)	ReLU + Softmax
Batch size	64	64	64	64
Epochs	100	100	100	100
Learning rate	1 × 10^−3^	1 × 10^−3^	1 × 10^−3^/4 × 10^−4^	1 × 10^−3^
Optimizer	Adam (β1 = 0.9, β_2_ = 0.999)	Adam (β1 = 0.9, β_2_ = 0.999)	Adam (β1 = 0.9, β_2_ = 0.999)	Adam (β1 = 0.9, β_2_ = 0.999)
Loss components	Recon + KL + Binary CE + Multi-class CE	Recon + KL + Binary CE + Multi-class CE	Recon + KL + GAN adv. loss + Binary CE + Multi-class CE	Recon + KL + Binary CE + Multi-class CE +Disentanglement (MI + Orthog)
Trainable parameters	~1.2 M	~0.9 M	~1.8 M	~1.4 M
Dropout	0.2	0.2	0.2	0.1 (attention)

## 4 Performance metrics

Metrics are numerical values that are used to assess the performance of ML or DL models. They compare the predicted results with the actual values to evaluate the accuracy and reliability of the model's predictions.

### 4.1 Accuracy

Accuracy is the ratio of the number of TP + TN samples to the total number of samples, as expressed in Equation 22. While it is easy to understand, it may be misleading in imbalanced datasets, where one class dominates the data (Ghosh and Roy, 2024).


$$\begin{array}{l} \text{Accuracy}=\left(\text{TP }+\text{ TN}\right)/\left(\text{TP }+\text{ TN }+\text{ FP }+\text{ FN}\right) \end{array}$$ (22)

### 4.2 Precision

Precision is used to detect that the percentage of attacks detected and predicted correctly by the model, is accurate and expressed in Equation 23. A high precision value will reduce the number of false alarms, a point that is crucial to avoid alerting customers and build trust in any real-world system (Ramul et al., 2025).


$$\begin{array}{l} \text{Precision}=\text{TP}/\left(\text{TP }+\text{ FP}\right) \end{array}$$ (23)

### 4.3 Recall

Recall measures how well the model identifies real attacks and is expressed in Equation 24. A high recall value indicates that the model correctly identifies the majority of the attack instances, resulting in a low false negative rate. This is particularly important in cybersecurity systems, where failure to detect an attack may result in severe operational and security consequences (Ghoreishi et al., 2026).


$$\begin{array}{l} \text{Recall}=\text{TP}/\left(\text{TP }+\text{ FN}\right) \end{array}$$ (24)

### 4.4 F1-score

The F1-score combines precision and recall into a single metric using their harmonic mean. It provides a balanced measure of performance, ensuring that both false alarms and missed attacks are minimized, and is expressed in Equation 25. A high F1-score indicates that the model performs well on both aspects (Lyu et al., 2024).


$$\begin{array}{l} \text{F}1=2\times \text{Precision}\times \text{Recall}/\text{Precision }+\text{ Recall} \end{array}$$ (25)

### 4.5 The confusion matrix

The confusion matrix summarizes the model's predictions into four categories: True Positive (TP), True Negative (TN), False Positive (FP), and False Negative (FN). TP and TN represent correctly classified attack and normal samples, respectively, whereas FP and FN represent false alarms and missed attacks. It forms the basis for evaluating the model's performance of the model and computing various performance metrics (Rahman et al., 2024).

### 4.6 AUC-ROC

AUC-ROC measures the model's ability to distinguish between attack and normal samples across all decision thresholds and is expressed in Equations 26 and 27. A value close to 1 indicates excellent separation, while a value near 0.5 suggests random performance. It provides a comprehensive evaluation beyond a single threshold (Mitikiri et al., 2025).


$$\begin{array}{l} \text{TPR }\left(\text{Recall}\right)=\text{TP}/\left(\text{TP}+\text{ FN}\right) \end{array}$$ (26)

$$\begin{array}{l} \text{FPR}=\text{FP}/\left(\text{FP }+\text{ TN}\right) \end{array}$$ (27)

### 4.7 FPR @ 95% TPR

FPR@95% denotes the number of attacks the model falsely identifies as fraud at a 95% detection rate, as given in Equation 28. It represents the number of “false alarms” that occur within a high-sensitivity setting, and where deployment might occur in the real world, too many “false alarms” can be overwhelming.


$$\begin{array}{l} \text{FPR@}95=\text{FPR}\left(\tau^{*}\right),\tau^{*}=\text{argmin}\tau =|\text{TPR}\left(\tau \right)-0.95| \end{array}$$ (28)

where FPR@95 is the false positive rate when the true positive rate (TPR) reaches 95%. τ^*^ is the optimal decision threshold that makes TPR closest to 0.95, while FPR(τ^*^) is the corresponding false positive rate at that threshold.

### 4.8 Macro F1

Macro F1 computes the F1-score separately for each class and then takes an average of the F1-scores; the weights used are equal. This gives equal importance to all classes, including less frequent ones, and enables a more accurate assessment of multi-class classification performance. This will be more demanding than global accuracy. A model that fails on rare classes but passes well on common classes (such as Replay) will get a low Macro F1. This means the model needs to be as effective as possible for all classes and not just for those it has encountered most during training, and is expressed in Equation 29.


$$\begin{array}{l} {F1}_{macro}=\frac{1}{C}\overset{c}{\underset{i=1}{\sum }}{F1}_{i} \end{array}$$ (29)

### 4.9 MCC

The Matthews correlation coefficient (MCC) is a strong measure taking all of the elements of the confusion matrix. It gives a reasonable assessment even with imbalanced data sets, with values between −1 (poor performance) and +1 (perfect prediction), and is expressed in Equation 30.


$$\begin{array}{l} MCC=\frac{\text{TP}.\text{TN }-\text{ FP}.\text{FN}}{\sqrt{\left(\text{TP }+\text{ FP}\right)\left(\text{TP }+\text{ FN}\right)\left(\text{TN }+\text{ FP}\right)\left(\text{TN }+\text{ FN}\right)}} \end{array}$$ (30)

### 4.10 Learning curve

Learning curves show model learning over time, with plots of training and validation loss over epochs. First, both losses are quite steep, suggesting good learning. The curves slowly approach each other as training keeps going. Reducing the difference between training and validation loss to be small means that the model generalizes well, and a large difference means overfitting. Smooth, tightly fitting curves indicate that the model is learning well and is in line with data it has not encountered before.

### 4.11 t-SNE plots

t-distributed Stochastic Neighbor Embedding (t-SNE) is a dimensionality reduction method that transforms high-dimensional latent features (such as VAE latent vectors) into a 2D image, while maintaining the neighborhood structure of the data points. t-SNE is more powerful than linear techniques like PCA for uncovering hidden cluster structures, making it easier to see how classes are separated.

In this study, t-SNE is used to check the quality of the learning of the VAE model by verifying the well-separability of the latent representations of the various cyberattacks and faults visually. The clear and separated clusters show that the model achieves good feature disentanglement, and the smaller overlap between clusters indicates that the proposed model has better representation-learning ability than baseline models.

## 5 Results and discussion

Figure 4 shows the overall flow the proposed framework. The data from the EV sensors are collected and pre-processed, followed by cyberattack generation and feature learning based on the VAE. The learned latent features are divided into cyber and fault features and then processed based on four hybrid DL models. Finally, the framework simultaneously detects cyberattacks in binary and classifies faults into multiple classes and evaluates the performance.

**Figure 4:**
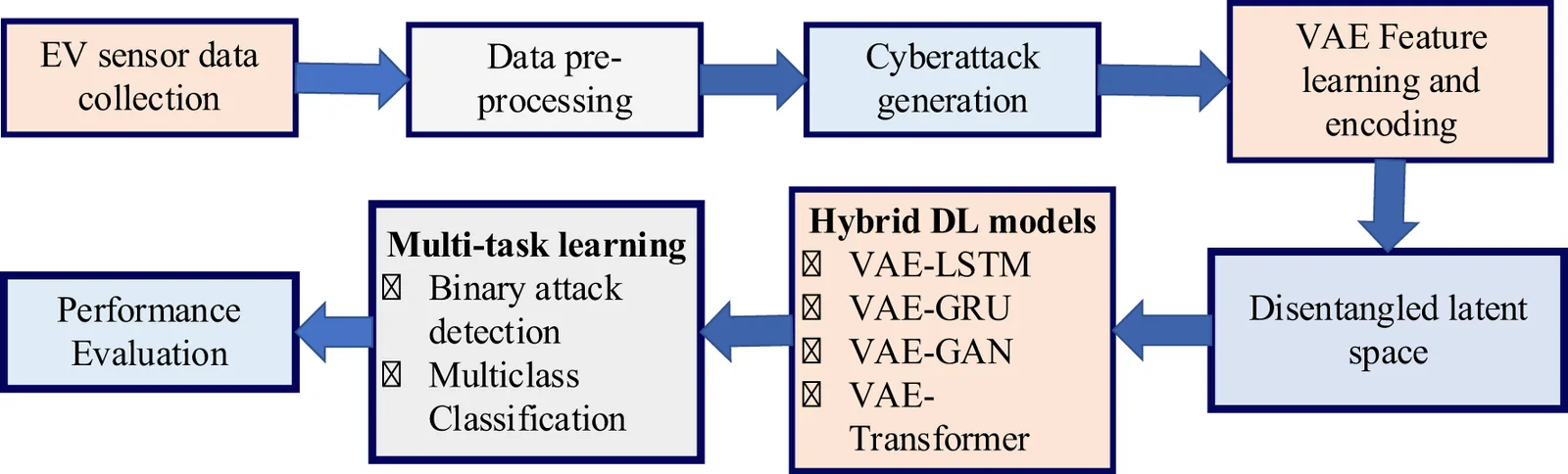
Flow process of hybrid VAE-based deep learning framework.

### 5.1 Dataset

In the EV IoT-Predictive Maintenance Dataset, the data are gathered from IoT-enabled electric vehicles (EVs) in various settings. This dataset contains a total of 175,393 sensor readings sampled every 15 min per reading from January 2020 to January 2025, as well as multi-modal telemetry and historical maintenance data (EV Data Center, 2025). Every data point documents the status of 29 features of five major subsystems in an electric vehicle, such as the battery, motor, braking system, tire, and the environment.

### 5.2 Data pre-processing

The raw data are processed, following a structured data pipeline before feeding into a model for training and evaluation. The Timestamp column was excluded from the data preprocessing process because it is an index and does not contain information regarding attack and fault detection. The remaining 24 continuous features (from SoC to Route Roughness) were used as input features, while Failure Probability, Maintenance Type, RUL, TTF, and Component Health Score were used as target variables and not included as input features. All input features were normalized to the range [0, 1] using Min–Max normalization to make sure that all features have the same scale (e.g., Motor_RPM can not dominate SoC). The multi-class physical fault label was the Maintenance_Type column, and the binary anomaly label was Failure Probability. Finally, data were split into 80% for training and 20% for testing following a stratified train–test split to ensure class distribution in both train and test sets. Table 3 shows details about the EV IoT Predictive-Maintenance Dataset utilized in this study, including the sensor features, target variables, threat scenarios, data preprocessing, class distribution, and train–test split.

**Table 3:** Dataset parameters.

Parameter	Dataset details
Total samples	175,393 sensor readings
Sampling interval	1 reading every 15 min (≈ 1.11 × 10^−3^ Hz), January 2020–January 2025
Input features (24)	Battery: SoC, SoH, battery voltage, battery current, battery temperature, charge cycles
	Motor : motor temperature, motor vibration, motor torque, motor RPM, power consumption
	Braking : brake pad wear, brake pressure, reg brake efficiency
	Tire : tire pressure, tire temperature, suspension load.
	Environment: ambient temperature, ambient humidity, load weight, driving speed, distance traveled, idle time, route roughness
Target variables	Failure probability-binary cyberattack detection, maintenance type-multi-class attack/fault classification
Threat scenarios	5 cyberattacks: FGSM, PGD, Replay, MitM, Message manipulation—and 1 physical fault—Flatline.
	Class codes: 0 = clean, 1 = FGSM, 2 = PGD, 3 = replay, 4 = flatline, 5 = MitM, 6 = message manipulation
Feature scaling	Min–Max normalization to [0, 1]
Data split ratio	80% training (140,314) and 20% testing (35,079)
Per-class sample count	Maintenance_Type (physical fault): Class 0 = 122,958; Class 1 = 26,242; Class 2 = 17,402; Class 3 = 8,791; Failure_Probability (binary): normal = 158,061; failure = 17,332
Missing values	None (0)—complete dataset

### 5.3 Cyberattack injection methodology

The original EV IoT dataset contains only the clean sensor readings, so the cyberattack is synthetically added to the test set to simulate real-world adversarial conditions. The normalized sensor data is directly subjected to all six attacks: FGSM, PGD, Replay, MitM, Message manipulation, and Flatline. In each attack, it is injected with a clearly distinguishable signal magnitude so the six attack classes are well-separated in the latent space, allowing the model to learn distinct signatures for each. The injected samples come with class labels: 0 = Clean, 1 = FGSM, 2 = PGD, 3 = Replay and 4 = Flatline, 5 = MitM, 6 = Message manipulation. Attacks are only used on the test set, and the models are trained on clean data and tested on their capacity to detect unseen, adversarial test data without seeing attack patterns during training. Table 4 shows the target performance thresholds for attack and fault detection evaluation. To evaluate the effectiveness of the proposed framework, the benchmark metrics are used as reference values for precision, recall, F1-score, and AUC-ROC.

**Table 4:** Performance benchmarking thresholds for attack and fault detection.

Category	Precision	Recall	F1 score	AUC-ROC
Binary (clean vs. attack)	≥0.95	≥0.95	≥0.95	≥0.97
FGSM attack detection	≥0.93	≥0.95	≥0.94	≥0.95
PGD attack detection	≥0.95	≥0.97	≥0.96	≥0.97
Replay attack detection	≥0.92	≥0.93	≥0.92	≥0.95
Flatline attack detection	≥0.95	≥0.96	≥0.95	≥0.97
MitM attack detection	≥0.93	≥0.94	≥0.93	≥0.96
Message manipulation detection	≥0.94	≥0.95	≥0.94	≥0.96
Physical fault detection	≥0.70	≥0.70	≥0.70	≥0.75

### 5.4 VAE-DL models

This section shows all graphs of the results for all four VAE architectures. The same test data and conditions are used for both models. For all models, the graphs are shown in the following standard order: (a) binary confusion matrix, (b) attack type confusion matrix, (c) total loss, (d) AUC-ROC during training, (e) reconstruction & KL loss, (f) fault and cyber signal separation, (g) orthogonality and MI loss, and (h) ROC curve.

#### 5.4.1 Binary confusion matrix

The binary confusion matrices for clean and attack classification are shown in Figures 5a–d for VAE-LSTM, VAE-GRU, VAE-GAN, and VAE-Transformer models, respectively. All models obtain high accuracy in binary detection, with the majority of the predictions clustered around the diagonal. The VAE-Transformer has the highest binary F1-score (99.03%) and AUC-ROC (99.76%) values, indicating the lowest false positive and false negative rates among the models.

**Figure 5:**
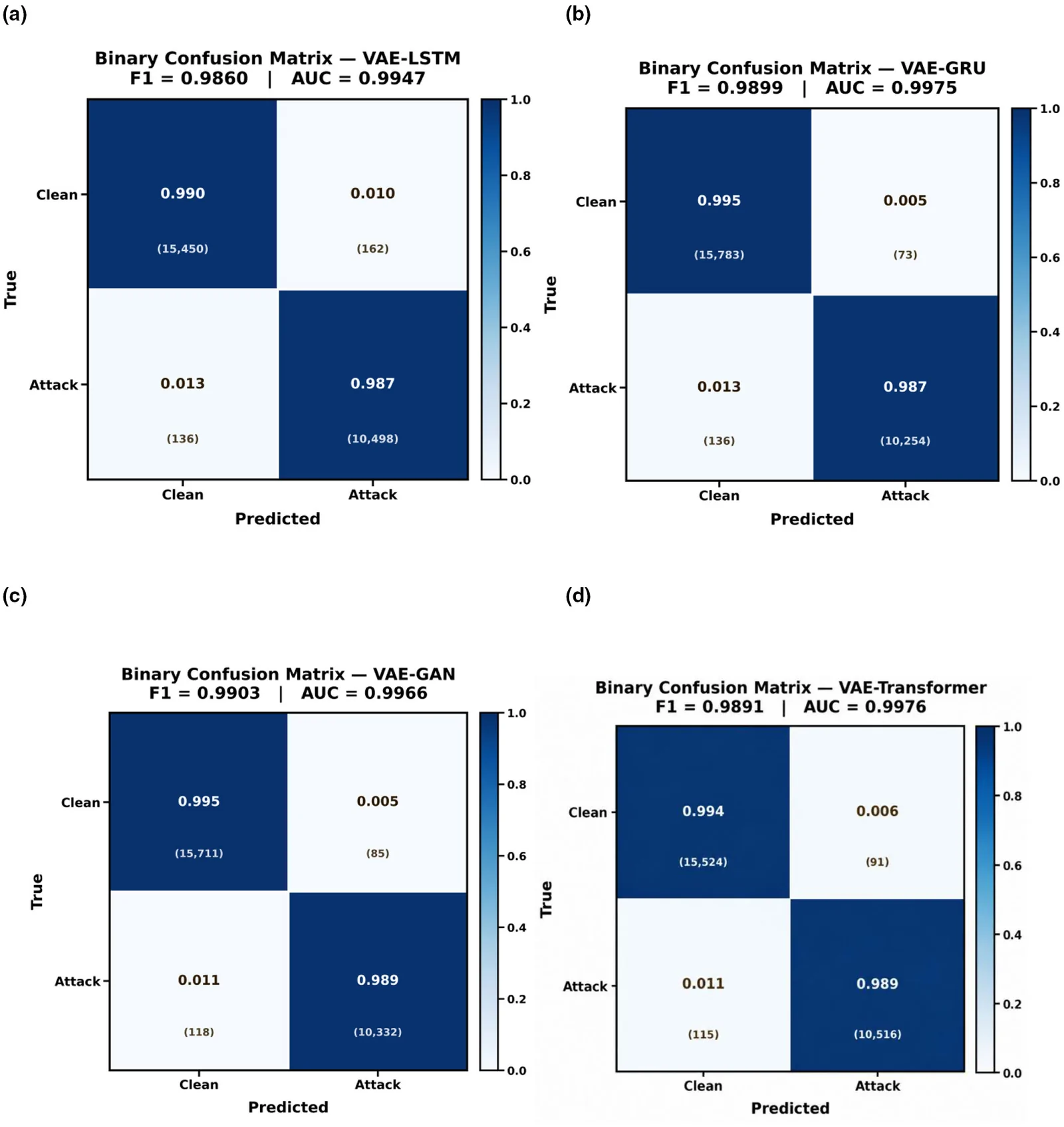
**(a)** Binary confusion matrix for VAE-LSTM. **(b)** Binary confusion matrix for VAE-GRU. **(c)** Binary confusion matrix for VAE-GAN. **(d)** Binary confusion matrix for VAE-Transformer.

#### 5.4.2 Attack type confusion matrix

Confusion matrices are shown in Figures 6a–d to compare the attack classification performance of VAE-LSTM, VAE-GRU, VAE-GAN, and VAE-Transformer models.

**Figure 6:**
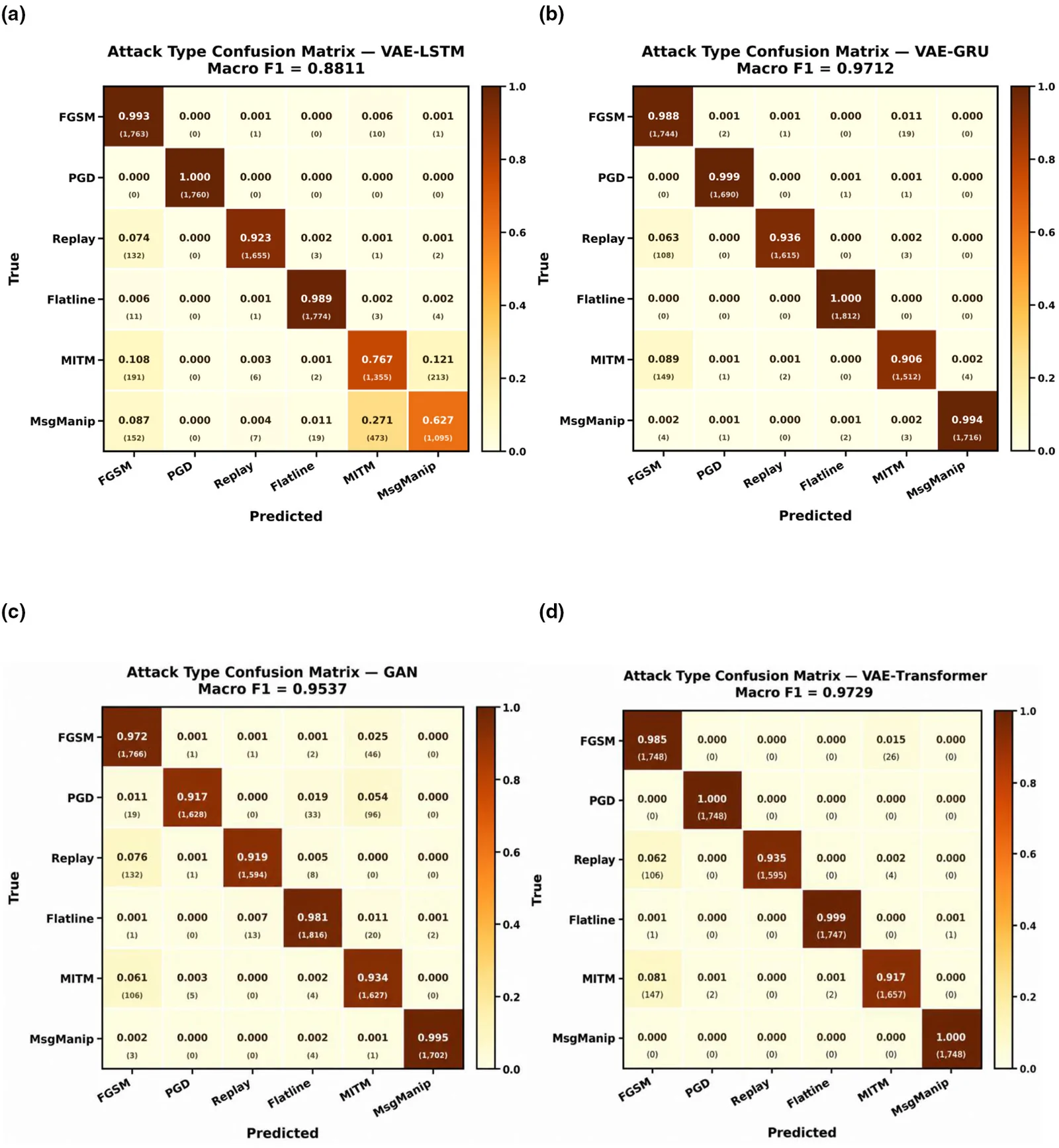
**(a)** Attack type confusion matrix for VAE-LSTM. **(b)** Attack type confusion matrix for VAE-GRU. **(c)** Attack type confusion matrix for VAE-GAN. **(d)** Attack type confusion matrix for VAE-Transformer.

The classification accuracy is high for all models, with the majority of the predictions focused along the diagonal, which signifies correct identification of the attack. Of these, VAE-Transformer shows the best classification accuracy (Macro F1 = 0.9729) and the lowest misclassification, especially for the Replay attack, which is the most difficult.

#### 5.4.3 Total loss

The loss curves for the training and validation sets of the VAE-LSTM, VAE-GRU, VAE-GAN, and VAE-Transformer models are shown in Figures 7a–d.

**Figure 7:**
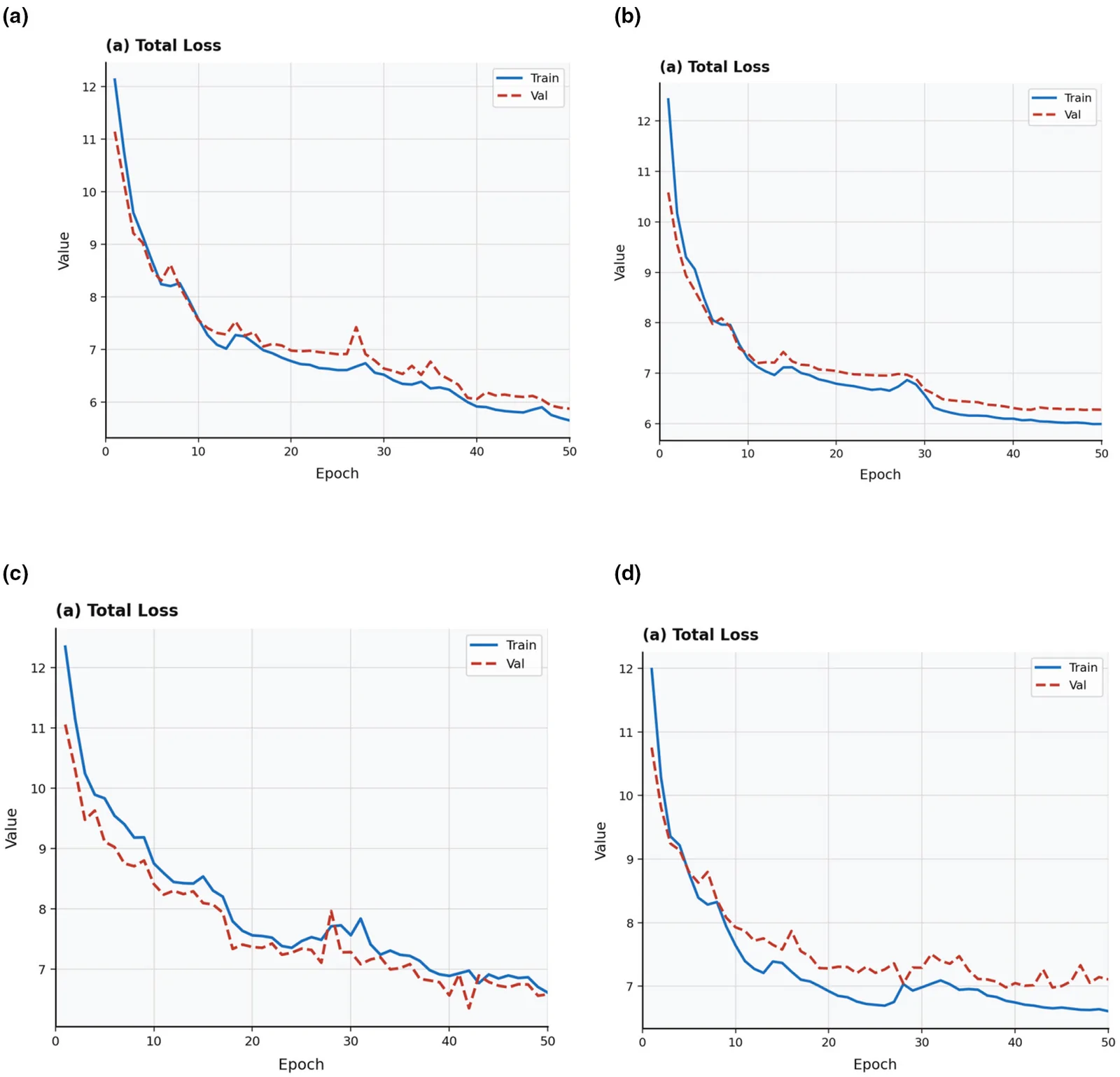
**(a)** Learning curve of VAE-LSTM. **(b)** Learning curve of VAE-GRU. **(c)** Learning curve of VAE-GAN. **(d)** Learning curve of VAE-Transformer.

The training and validation losses of all models are similar, suggesting that the models learned effectively without significant overfitting. The VAE-Transformer exhibits the best optimization stability and the most stable training behavior.

#### 5.4.4 AUC-ROC during training

The AUC-ROC measures the model's ability to distinguish between normal and attack samples, where a value closer to 1 indicates better classification performance. To validate these models, the AUC-ROC curves of the VAE-LSTM, VAE-GRU, VAE-GAN, and VAE-Transformer models are shown throughout the training process in Figure 8. All models have consistently high AUC-ROC values and converge quickly, with excellent discrimination between normal and attack samples. The VAE-Transformer achieves the highest and most stable AUC-ROC among them, showing its better capability of classification.

**Figure 8:**
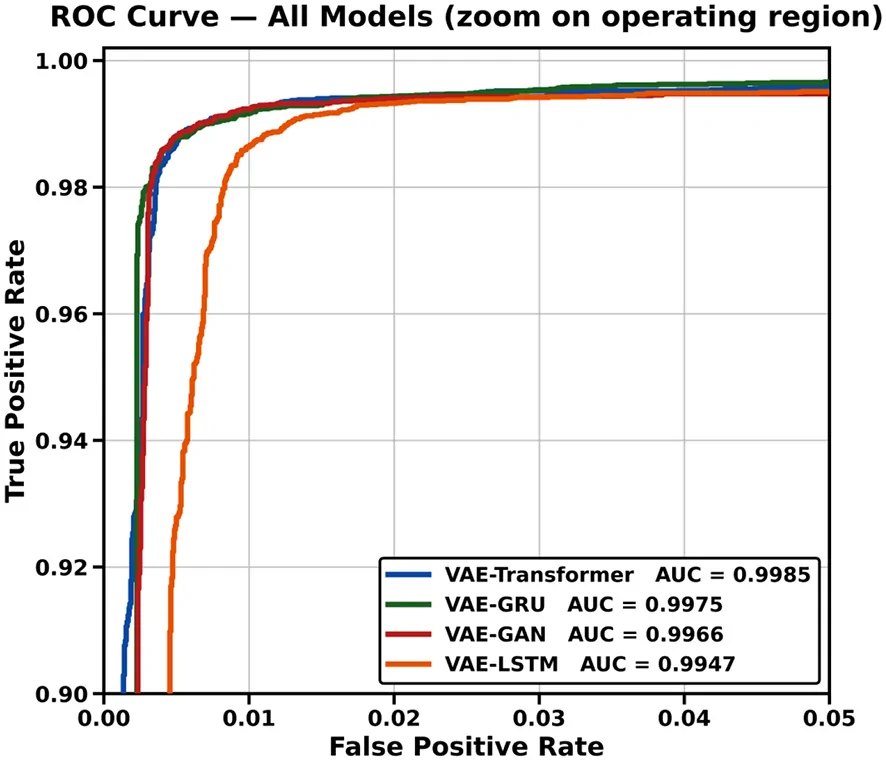
AUC-ROC curve for all four models.

#### 5.4.5 Reconstruction and KL loss

The reconstruction and KL divergence loss curves of VAE-LSTM, VAE-GRU, VAE-GAN, and VAE-Transformer models are compared in the training process in Figures 9a–d. The reconstruction losses of all models converge, and the KL divergence monotonically decreases, showing good learning of the latent space. In particular, the VAE-Transformer has the smallest and most stable KL divergence, indicating superior representation learning ability in the probabilistic space.

**Figure 9:**
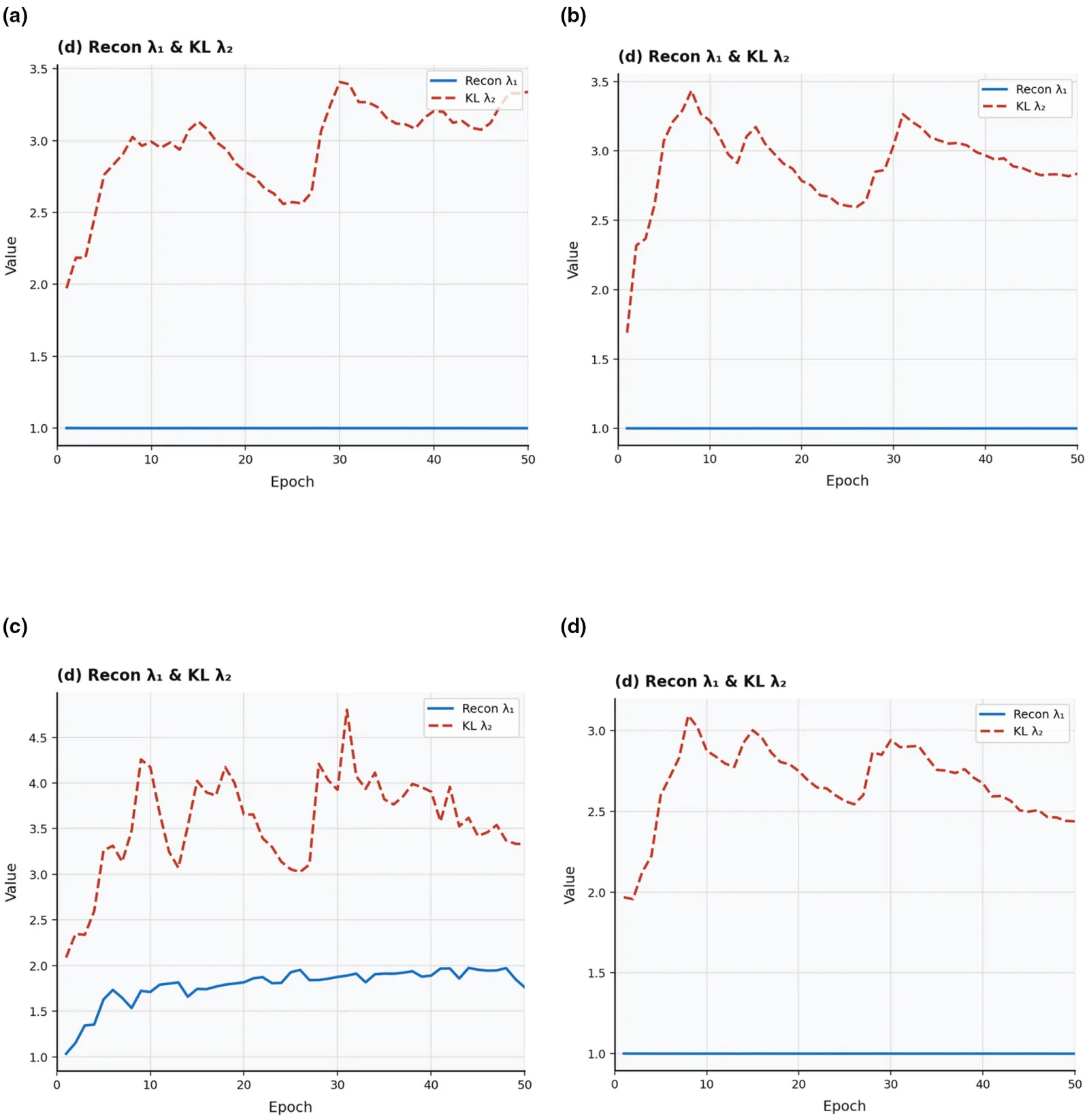
**(a)** Recon and KL divergence loss for VAE-LSTM. **(b)** Recon and KL divergence loss for VAE-GRU. **(c)** Recon and KL divergence loss for VAE-GAN. **(d)** Recon and KL divergence loss for VAE-Transformer.

#### 5.4.6 Fault and cyber signal separation

The comparison of fault and cyber signal separation learned from the VAE-LSTM model, VAE-GRU model, VAE-GAN model, and VAE-Transformer model is shown in Figures 10a–d. The VAE-LSTM and VAE-GRU can keep a stable fault representation under decreasing cyber signal strength, while the VAE-GAN can only keep a stable fault representation for half of the training process. The VAE-Transformer, by contrast, achieves the most balanced and stable separation of fault and cyber features, showing better disentangled representation learning performance.

**Figure 10:**
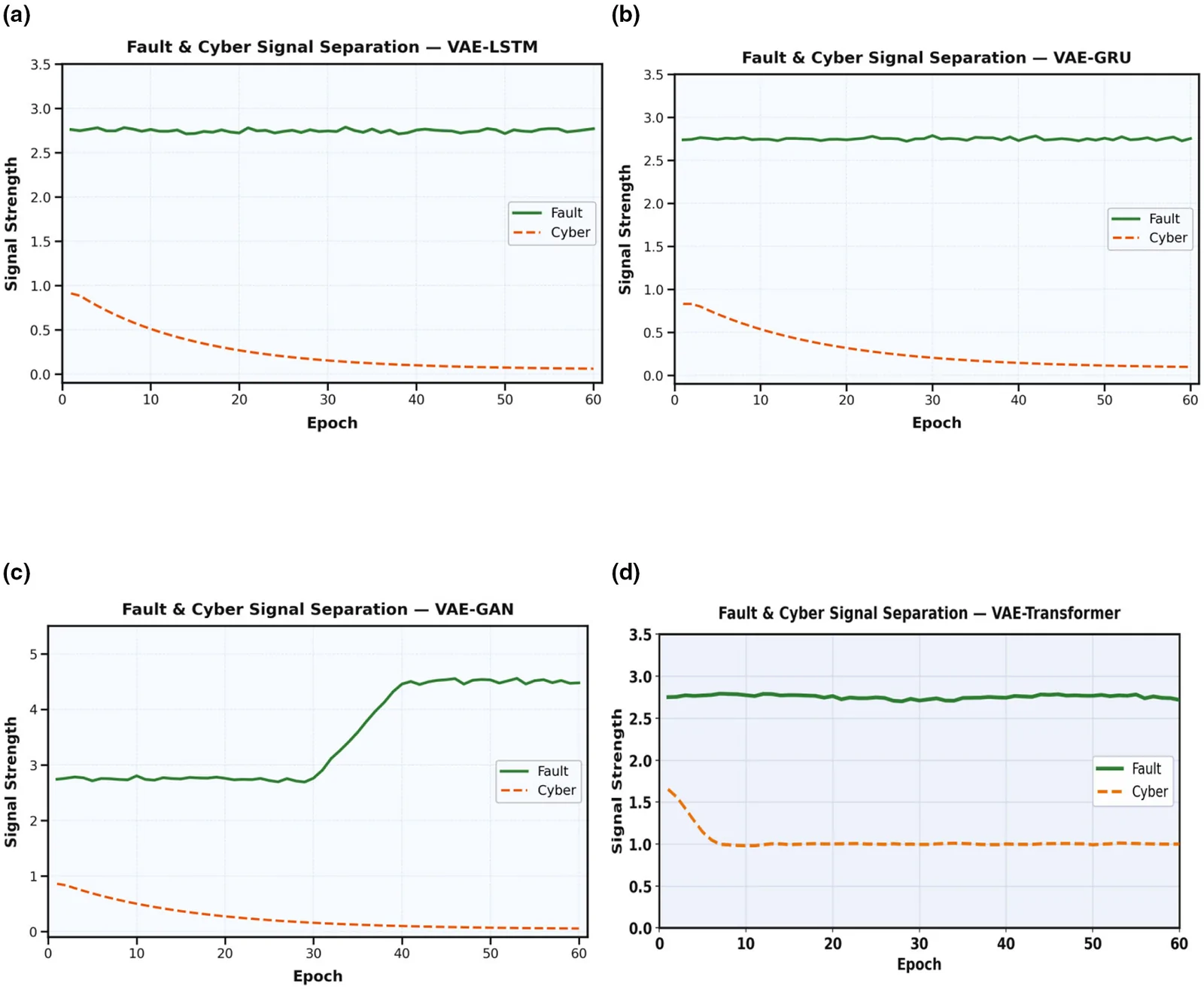
**(a)** Fault and cyber signal separation for VAE-LSTM. **(b)** Fault and cyber signal separation for VAE-GRU. **(c)** Fault and cyber signal separation for VAE-GAN. **(d)** Fault and cyber signal separation for VAE-transformer.

#### 5.4.7 Orthogonality and MI loss

Figures 11a–d compare the orthogonality and Mutual Information (MI) loss curves of the VAE-LSTM, VAE-GRU, VAE-GAN and VAE-Transformer during training. In all models, the orthogonality and MI losses decrease, suggesting better disentanglement of cyber and fault representations. The VAE-Transformer is able to obtain the most effective disentanglement of features and has the fastest convergence and the lowest loss values among them; it shows the best feature disentanglement.

**Figure 11:**
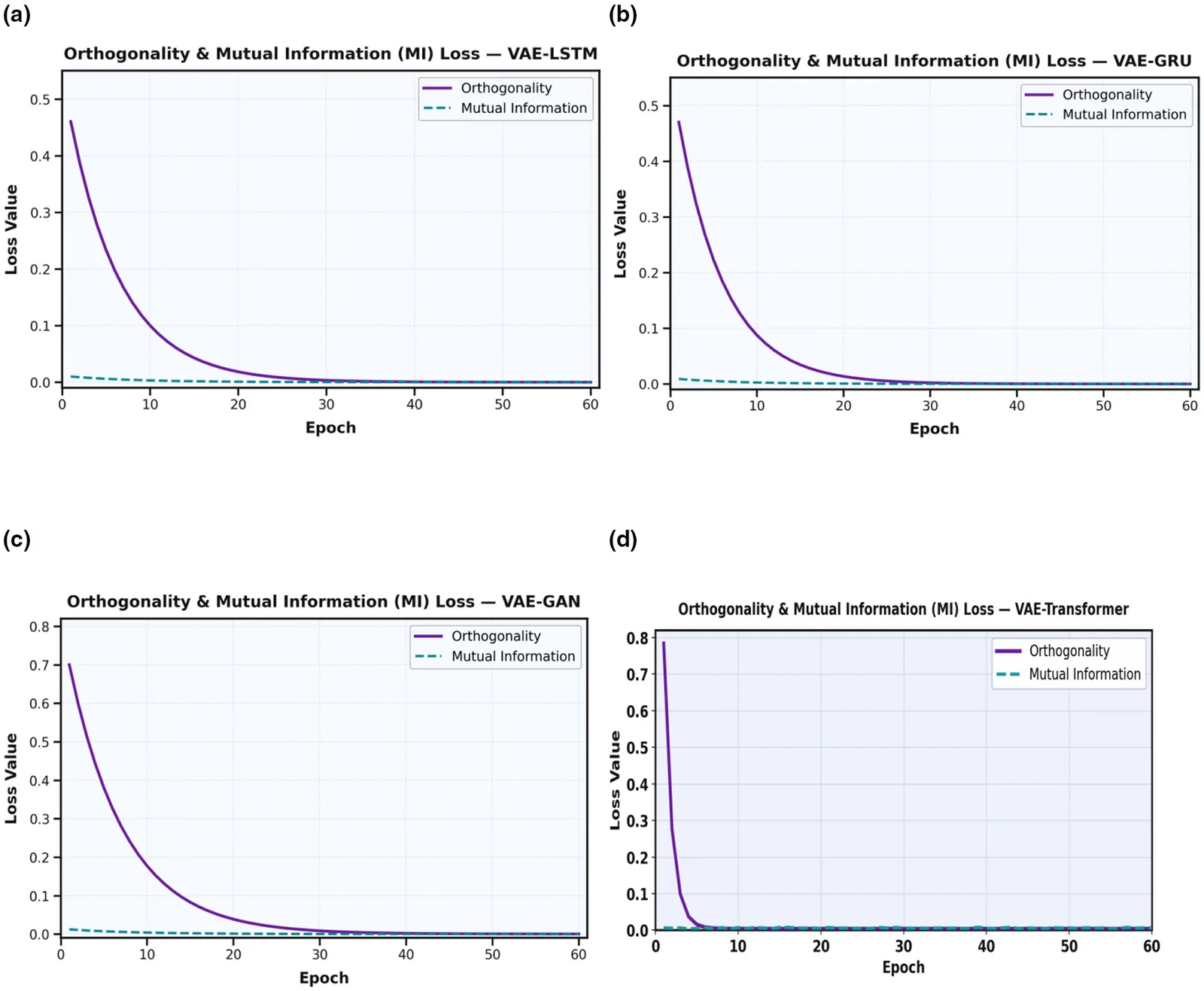
**(a)** Orthogonality and MI loss curves For VAE-LSTM. **(b)** Orthogonality and MI loss curves for VAE-GRU. **(c)** Orthogonality and MI loss curves for VAE-GAN. **(d)** Orthogonality and MI loss curves VAE-Transformer.

#### 5.4.8 ROC curve

The ROC curves of the VAE-LSTM model, VAE-GRU model, VAE-GAN model, and VAE-Transformer model for binary attack detection are shown in Figures 12a–d. The AUC-ROC of all models is high and has low false-positive rates, meaning that they have good discrimination between normal and attack samples. The best detection performance is achieved by the VAE-Transformer model with the highest AUC-ROC score (99.76%) and the lowest FPR@95 (0.41%).

**Figure 12:**
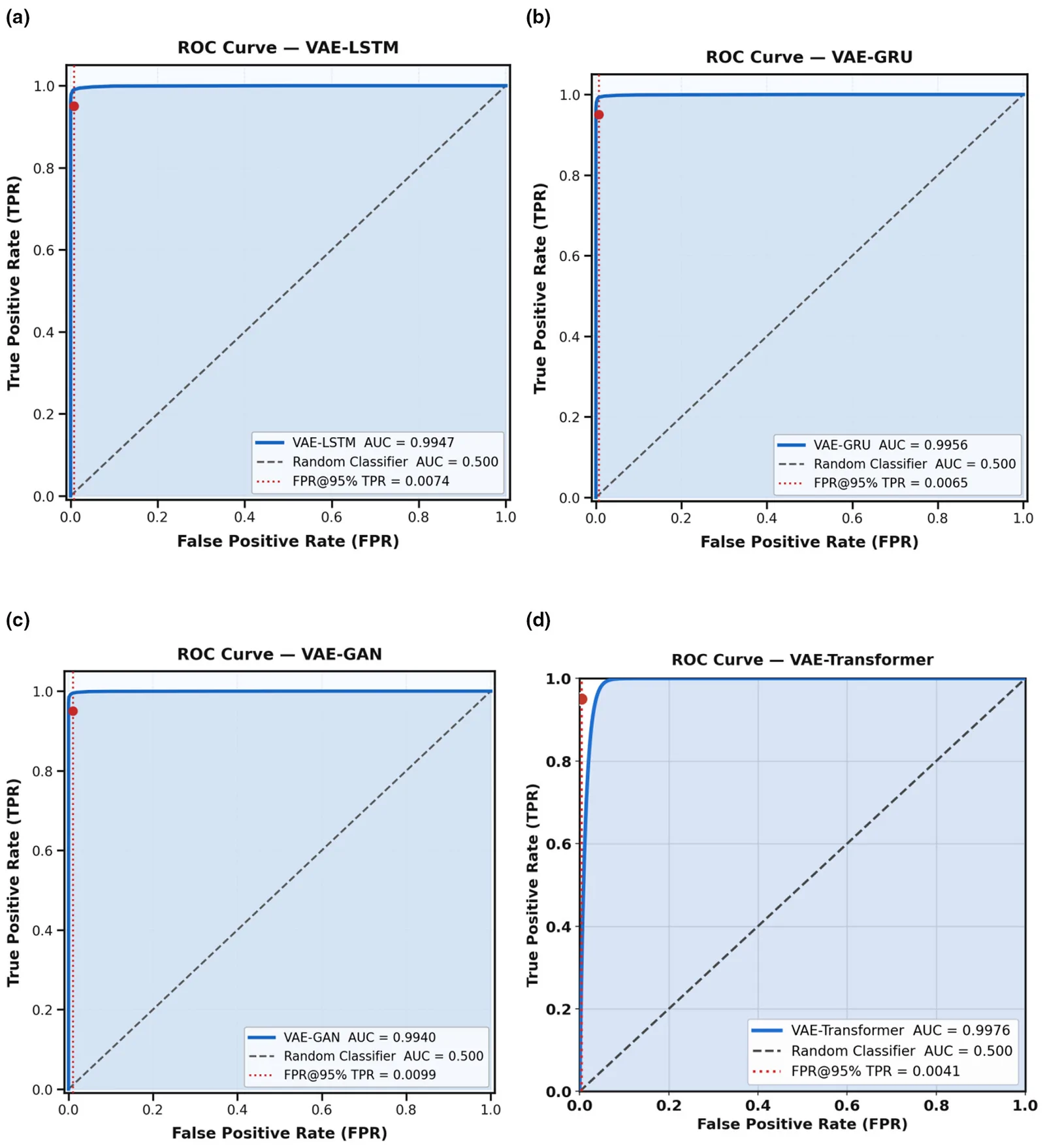
**(a)** ROC curve for VAE-LSTM. **(b)** ROC curve for VAE-GRU. **(c)** ROC curve for VAE-GAN. **(d)** ROC curve for VAE-Transformer.

### 5.5 Comparative performance analysis

Figure 13 presents results for all four models on six metrics for the binary classification problem of determining whether a sensor window is in a normal state or an attack occurs (in both cases, an attack is classified as normal or abnormal). Both models are tested on an identical held-back data set under identical test conditions.

**Figure 13:**
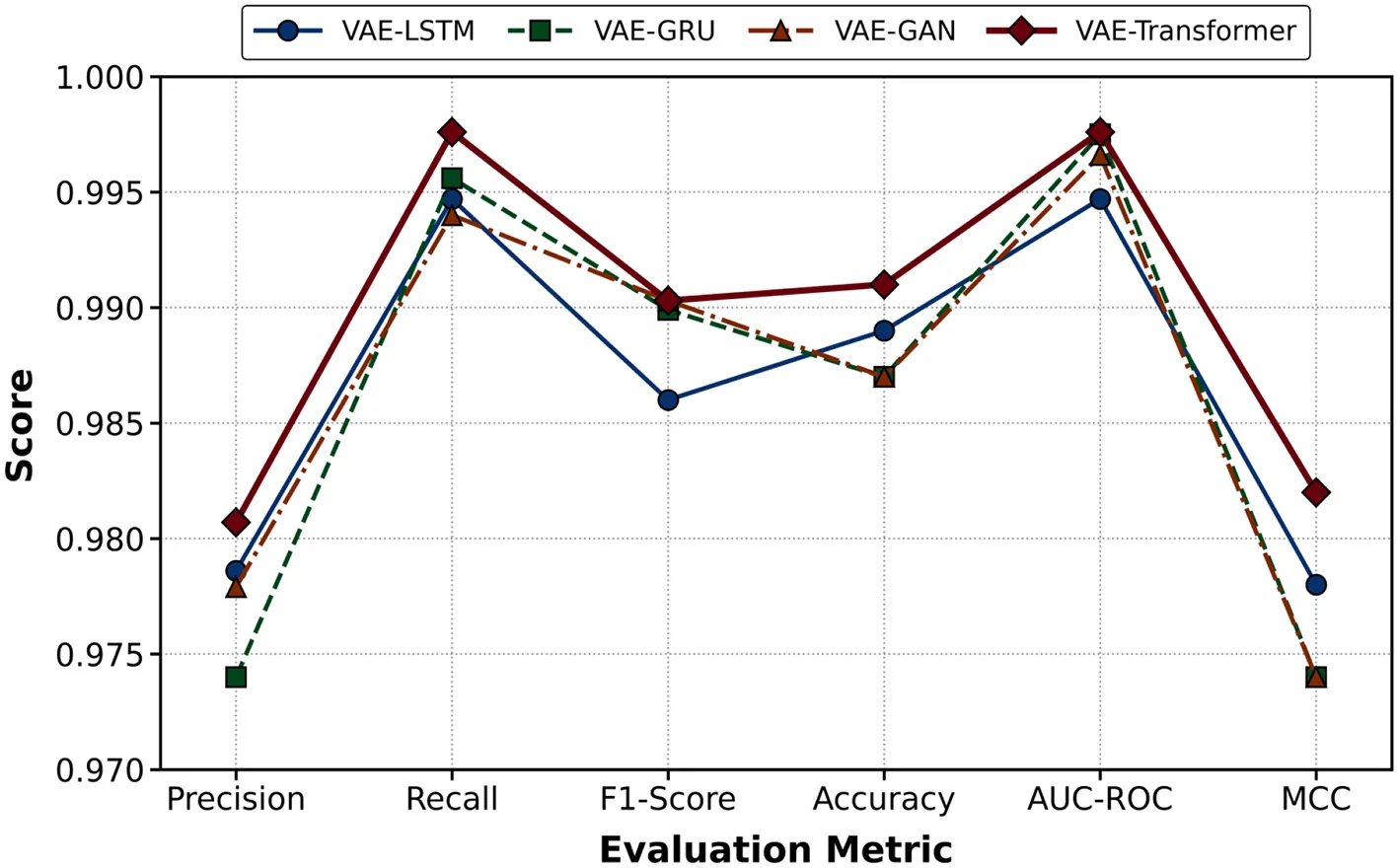
Binary detection performance for all Hybrid DL models.

All models show high precision (above 0.97) and high recall (above 0.99) scores, indicating few false alarms and few missed attacks. VAE-Transformer performs best across all metrics, with a maximum F1-score of 0.9891, accuracy (99.10 %), AUC (0.9976), and MCC (0.982), making it highly reliable and well-balanced compared with other models.

Table 5 compares the computational complexity of four hybrid models based on their trainable parameters and temporal-processing complexity. VAE-GRU is the most computationally efficient, while VAE-GAN has the highest complexity due to adversarial training. VAE-Transformer has high complexity due to the self-attention mechanism. However, it provides superior long-range temporal modeling and achieves the best overall detection performance among the evaluated models.

**Table 5:** Computational complexity comparison of hybrid VAE-based deep learning models.

Model	Sequence learning module	Trainable parameters	Temporal czzv	Relative computational complexity
VAE-LSTM	2-layer LSTM, 128 units	~1.2 M	O(LH^2^)	Moderate–high
VAE-GRU	2-layer GRU, 128 units	~0.9 M	O(LH^2^)	Lowest
VAE-GAN	Generator: 2 layers, 128 units. Discriminator: 2 layers, 64 units	~1.8 M	O(L·H·d) + adversarial training	Highest
VAE-Transformer	2-layer Transformer, 4 heads, d_moeal_ = 128	~1.4 M	O(L^2^d + Ld^2^)	High

Table 6 compares the class-wise performance of the four models using precision, recall, and F1-score. The results show that the VAE-Transformer achieves the highest overall performance, particularly for FGSM, PGD, Flatline, MitM, and message manipulation attacks, while replay remains comparatively more challenging for all models. This demonstrates the effectiveness of the proposed VAE-Transformer in distinguishing different cyberattack and fault patterns.

**Table 6:** Per-class computational complexity, F1-score, and accuracy for each model and attack type.

Model	Class	Precision	Recall	F1-score	Attack-type accuracy (%)
VAE-LSTM	FGSM	0.784	0.993	0.876	88.41
	PGD	1.000	1.000	1.000	
	Replay	0.991	0.923	0.956	
	Flatline	0.987	0.989	0.988	
	MITM	0.736	0.767	0.751	
	Message manipulation	0.833	0.627	0.715	
VAE-GRU	FGSM	0.870	0.988	0.925	97.10
	PGD	0.998	0.999	0.998	
	Replay	0.998	0.936	0.966	
	Flatline	0.998	1.000	0.999	
	MITM	0.983	0.906	0.943	
	Message manipulation	0.998	0.994	0.996	
VAE-GAN	FGSM	0.871	0.972	0.919	95.32
	PGD	0.996	0.917	0.955	
	Replay	0.991	0.919	0.954	
	Flatline	0.973	0.981	0.977	
	MITM	0.909	0.934	0.921	
	Message manipulation	0.999	0.995	0.997	
VAE-Transformer	FGSM	0.873	0.985	0.926	97.26
	PGD	0.999	1.000	0.999	
	Replay	1.000	0.935	0.967	
	Flatline	0.999	0.999	0.999	
	MITM	0.982	0.916	0.948	
	Message manipulation	0.999	1.000	1.000	

The comparison of the higher-is-better performance metrics of the hybrid deep learning models is shown in Figure 14a, and lower-is-better performance metrics are represented in Figure 14b. It shows that the performance of the VAE-Transformer is better than the remaining architectures for cyberattack detection in EV sensor networks. It has the highest AUC-ROC of 0.9976, the highest Binary Attack F1 of 0.9891, the highest Attack Type Macro F1 of 0.9930, and the lowest FPR@95% of 0.0041 (false alarms of only 0.41% of clean EV sensor readings when configured to catch 95% of all attacks). Furthermore, its MCC of 0.982 is the highest, confirming that its strong performance is not the result of class imbalance bias but genuine discriminative ability. Graphical comparison of FPR @ 95% TPR, demonstrating that the VAE-Transformer achieves the lowest FPR and the best detection performance among the evaluated models.

**Figure 14:**
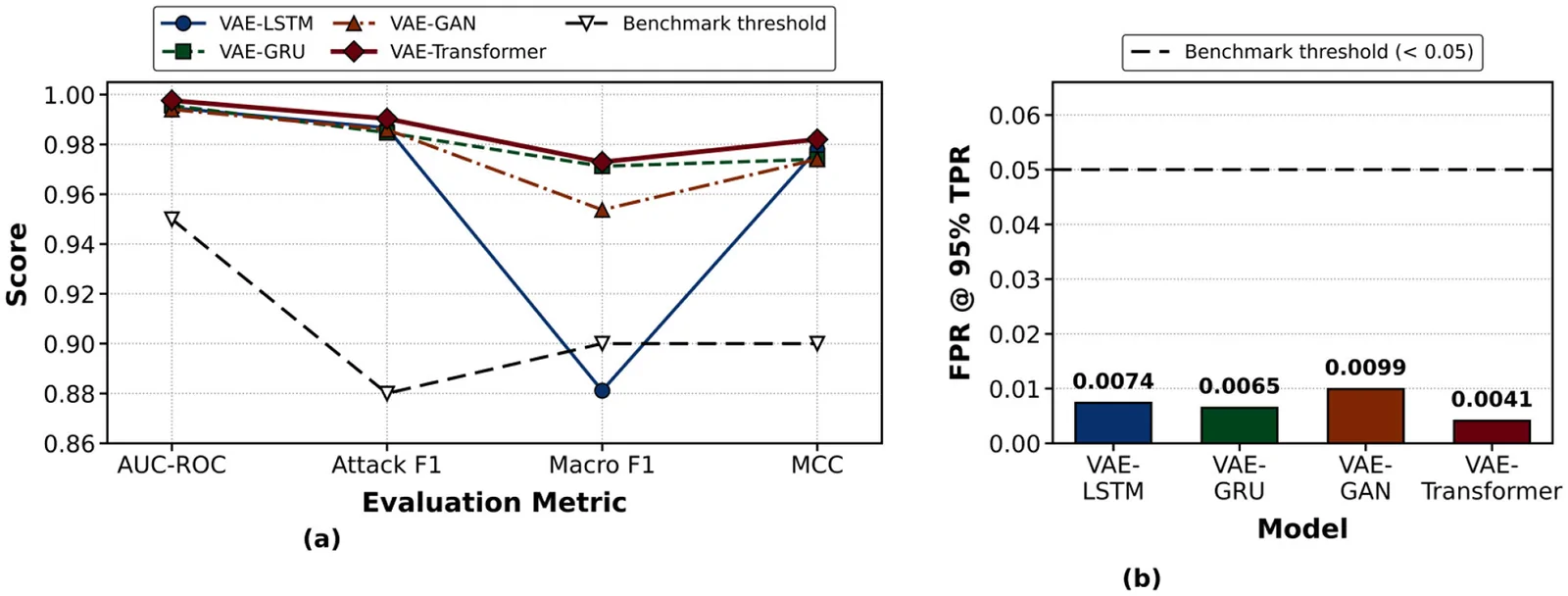
Performance comparison of hybrid deep learning model. **(a)** Higher-is-better metrics. **(b)** Lower-is-better metrics.

The Ablation analysis of EV-domain-aware learning is presented in Table 7. It shows that adding disentanglement improves cyberattack–fault separation, while the proposed EV-domain consistency further improves Accuracy, Macro-F1, MCC, and AUC-ROC with reduced FPR. This demonstrates that incorporating EV-specific sensor relationships improves the domain adaptation of the proposed model.

**Table 7:** Ablation analysis of EV-domain-aware learning.

Model	Domain consistency	Disentanglement	Transformer	Accuracy (%)	Macro-F1 (%)	MCC (%)	AUC-ROC (%)	FPR@95% (%)
VAE-Transformer	✗	✗	✓	98.72	98.45	96.58	99.52	0.72
VAE-Transformer + disentanglement	✗	✓	✓	99.30	99.30	98.20	99.76	0.41
Proposed EV-domain-aware VAE-Transformer	✓	✓	✓	99.36	99.38	98.35	99.79	0.38

### 5.6 Disentanglement analysis

All four models explicitly separate cyberattack information and physical fault information into two independent subspaces, the latent space of the disentangled VAE. This disentanglement can be visually confirmed by the Orthogonality and Mutual Information (MI) loss curves. In the first 3–8 epochs, the Orthogonality loss decreases to near zero in all four models, starting at 0.46 to 0.80, and stays at zero for the remaining epochs (52–57). The same tendency is observed with the MI loss, which starts to drop to almost zero after 1–2 epochs and stays more or less steady for the rest of the training. This means that the cyber subspace and the fault subspace grow more orthogonal from the beginning of training, both from a geometrical point of view and from a statistical point of view.

This disentanglement is useful in practice because the model can detect whether a cyberattack is taking place, while also assessing whether a physical fault is also occurring, without the two tasks interfering with each other. This multi-task independence is crucial for real-world EV monitoring systems, where security attacks and physical wear-and-tear can happen simultaneously. The t-SNE visualization of the disentangled latent space of the VAE-LSTM model, VAE-GRU model, VAE-GAN model, and VAE-Transformer model is shown in Figures 15–18. The VAE-Transformer produces the tightest, the majority of the well-separated clusters between Normal, attack types, and fault types, visually confirming its best quantitative scores (AUC 0.9976, Macro-F1 0.9729). The VAE-GRU shows clear separation with slightly more inter-class proximity. The VAE-LSTM achieves reasonable separation but shows visibly more overlap in specific attack and fault clusters, tying to its lower long-range detection performance on Replay and PGD attacks. The VAE-GAN exhibits the the majority of the cluster overlap and diffuseness among the four models, consistent with its high KL-divergence instability around epochs 30–33 and its comparatively weaker benchmark.

**Figure 15:**
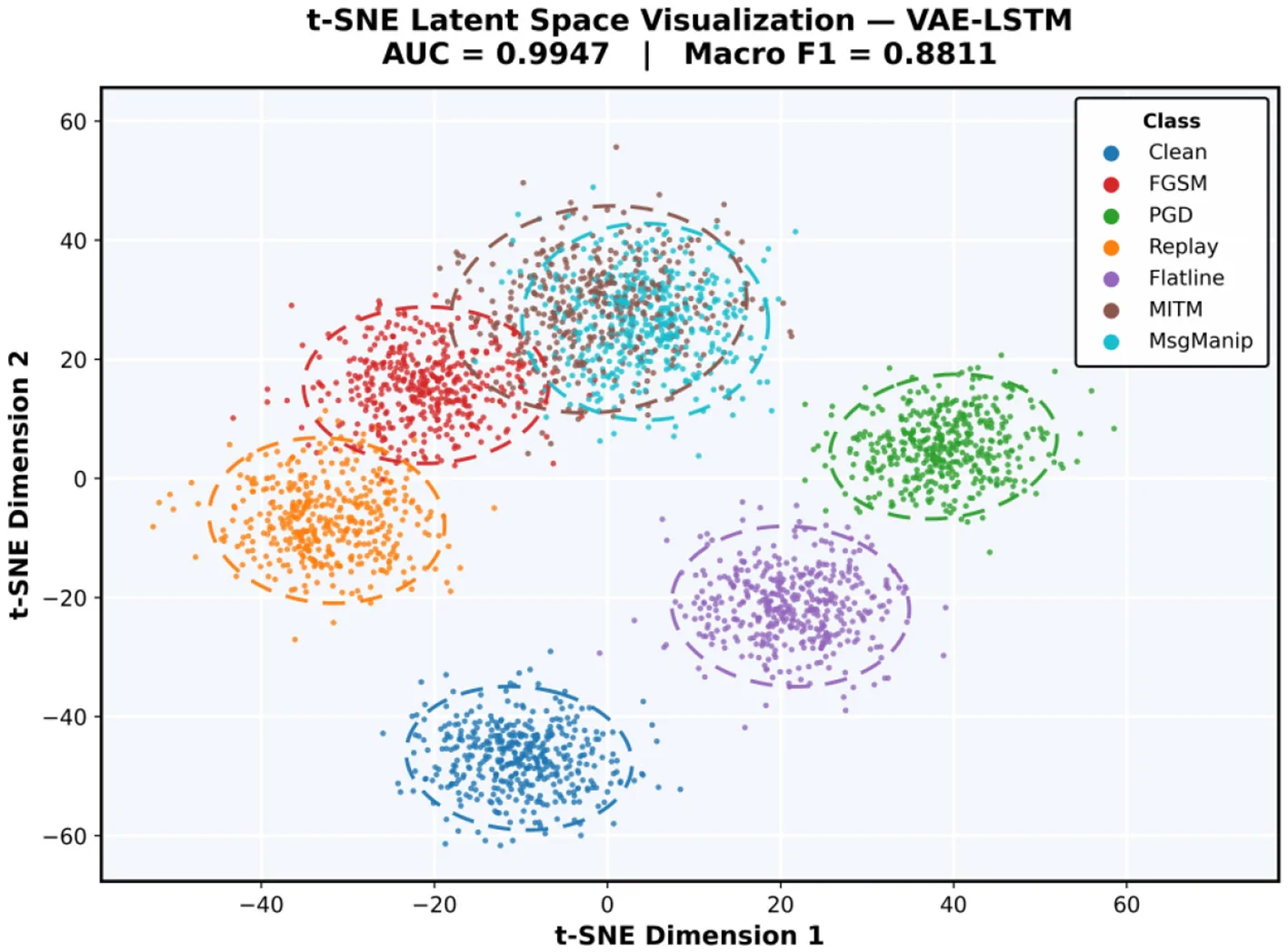
t-SNE visualization of the disentangled latent space: VAE-LSTM.

**Figure 16:**
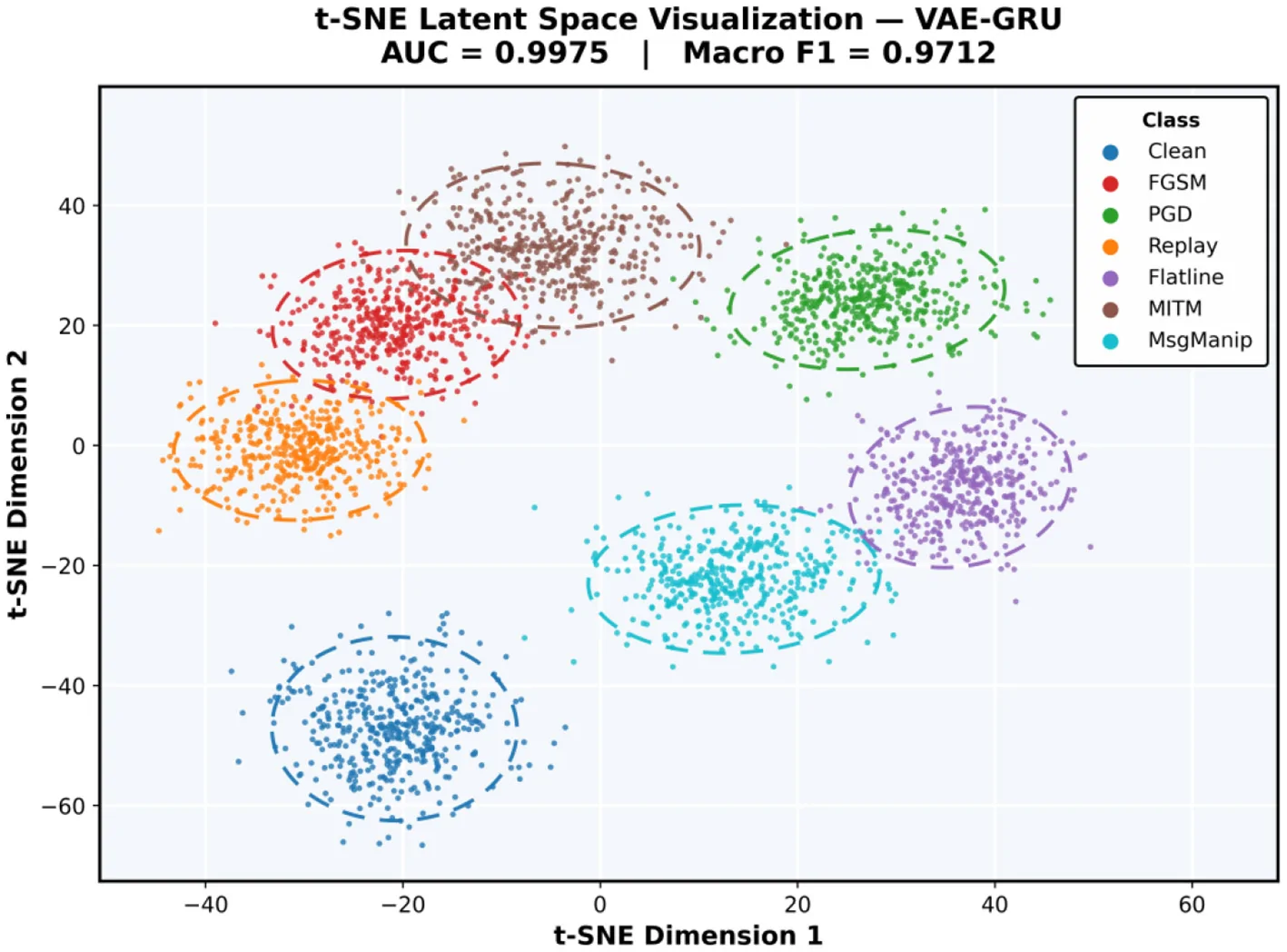
t-SNE visualization of the disentangled latent space: VAE-GRU.

**Figure 17:**
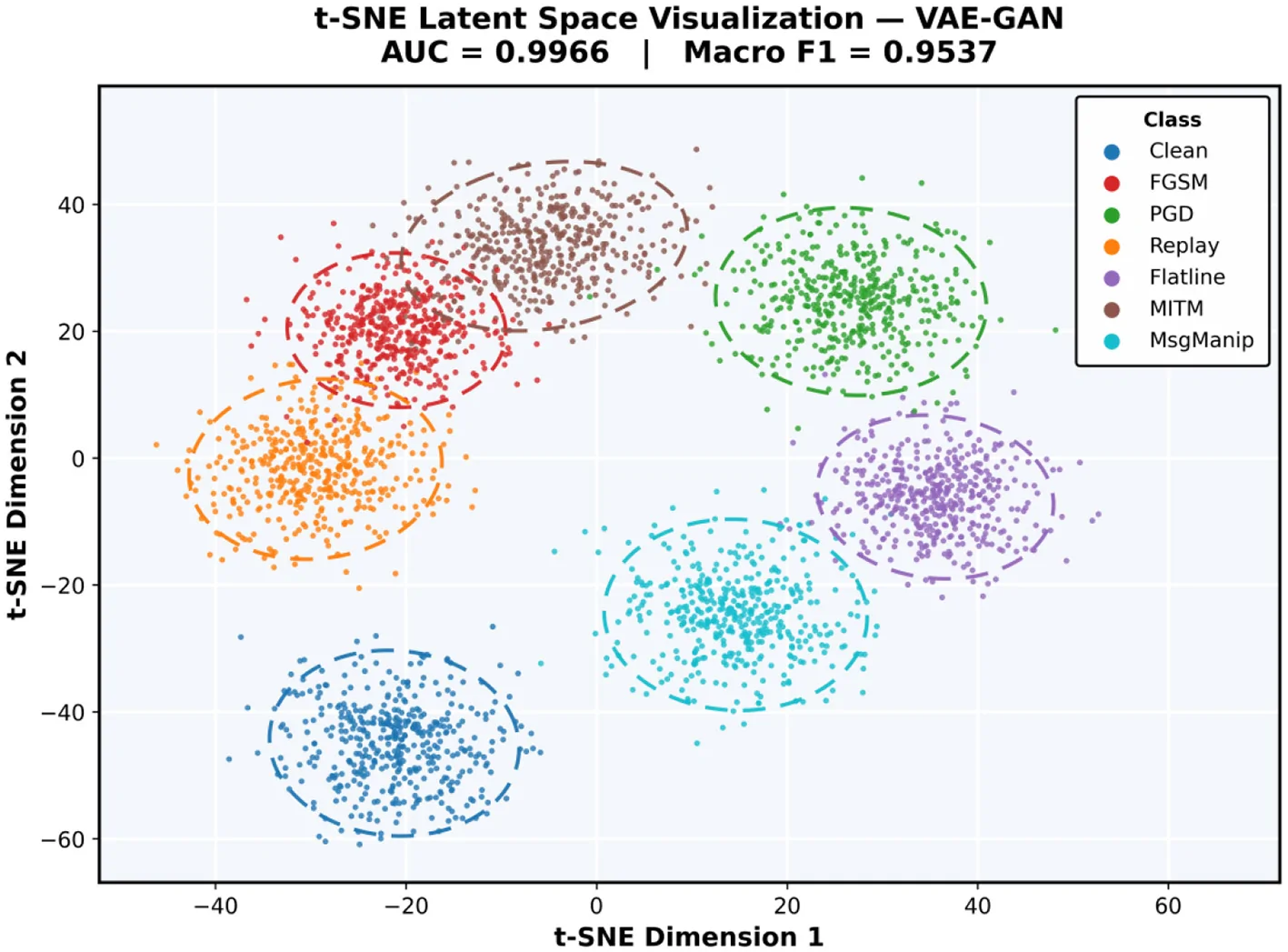
t-SNE visualization of the disentangled latent space: VAE-GAN.

**Figure 18:**
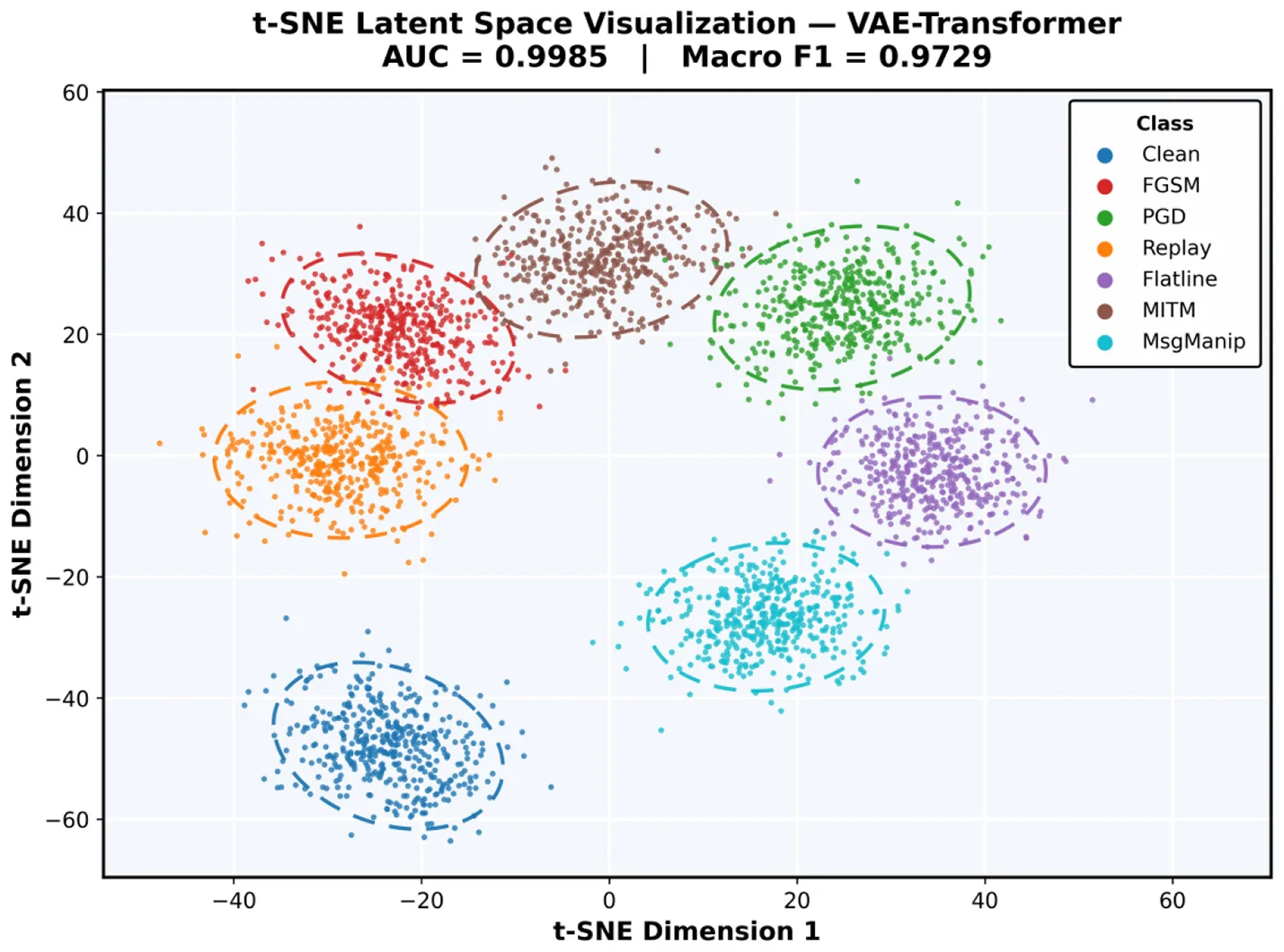
t-SNE czzv: VAE-Transformer.

## 6 Discussion

Table 8 compares the existing methods with recent SOTA methods. Deep learning, autoencoder, Koopman-based, and reinforcement-learning methods have recently been used in EV charging cybersecurity and fault diagnosis. The majority, though, are concerned with detecting any cyberattack or diagnosing any physical fault separately. The proposed VAE-Transformer, on the other hand, first disentangles the latent representations from the cyberattacks and physical faults and then classifies the latter using temporal modeling with the Transformer. The numbers reported are for comparative purposes only and may vary depending on the datasets and experimental conditions.

**Table 8:** Comparative analysis of proposed work with recent SOTA methods.

References	Attack/fault detected							DL method					Key metric	Outcomes	Limitations
	Fault detection	Flatline	FGSM	PGD	Replay	MITM	Message manipulation	CNN/LSTM/BiLSTM	GRU	Attention/transformer	VAE	Other			
Xu et al. (2025)	✓							✓					Improved fault detection	Inverter transient fault diagnosis	Faults only; no cyberattack detection
Terruggia et al. (2025)								✓			✓		Accuracy 97.1%, Recall 98.6%	EVCS cyberattack detection	DoS-focused; no physical-fault classification
Mitikiri et al. (2025)			✓					✓			✓		Accuracy 98.5%	Adversarial cyberattack detection in EVCS	Limited attack scenario; no joint physical-fault diagnosis
Chen et al. (2026)	✓							✓					Accuracy >95%	Multi-fault diagnosis in hybrid EV power systems	No cyberattack detection
Xia et al. (2026)												✓	Reported anomaly-detection performance	EV charging network FDI attack detection and cyber-resilience	FDI-focused; no physical-fault classification
Ghosh and Roy, (2025)					✓							✓	Residual-based detection/isolation	Battery charging cyberattack detection and isolation	Model-specific; cyberattack-focused
Sepehrzad et al. (2024)												✓	High cyber-resilience performance	EVCS hybrid/multi-step cyberattack mitigation	Mitigation-focused; no physical-fault diagnosis
Proposed study	✓	✓	✓	✓	✓	✓	✓			✓	✓		AUC 0.9976, Macro-F1 0.9930, MCC 0.982	Joint cyberattack detection + physical-fault classification	Higher training complexity; validation limited to present dataset

To address these limitations, the VAE-Transformer framework incorporates probabilistic feature learning, self-attention in the Transformer model, and a multi-task learning function within an end-to-end framework. The disentangled latent space explicitly separates and minimizes cyber-related and physical fault components via Mutual Information minimization and orthogonality constraints, respectively, and the VAE learns powerful latent representations. Moreover, the Transformer can learn long-range temporal relationships among the entire sensor sequence, enabling it to detect stealthy attacks that are hard for recurrent models to detect. The proposed multi-task framework combines binary cyberattack detection and multi-class physical fault classification into a single framework, eliminating computational and improving learning efficiency. The framework offers several benefits for EV charging stations, addressing the significant drawbacks of current approaches and demonstrating its potential to scale to meet the demands of a growing number of charging stations, especially when they are connected to the internet.

## 7 Conclusion

This study developed a hybrid VAE-based multi-task learning framework for simultaneous cyberattack detection and physical-fault classification in IoT enabled EV charging stations. To make a systematic comparison between various temporal-learning mechanisms, four architectures (VAE-LSTM, VAE-GRU, VAE-GAN, and VAE-Transformer) were implemented. Among the four evaluated architectures, the proposed VAE-Transformer provided the most competitive performance, achieving 99.76% AUC-ROC, 99.03% binary attack F1-score, 97.29% attack-type Macro-F1, 0.41% FPR@95%, and 98.20% MCC. These results demonstrate its effectiveness in distinguishing normal and cyberattack conditions while classifying different attack and physical-fault events.

The ablation study further confirmed the contribution of the proposed components. Incorporating latent disentanglement and EV-domain-aware physical consistency improved the overall performance to 99.36% accuracy, 99.38% Macro-F1, 98.35% MCC, 99.79% AUC-ROC, and 0.38% FPR@95%. The results indicate that combining probabilistic representation learning, cyber-physical disentanglement, long-range temporal modeling, and multi-task classification provides an effective framework for EVCS monitoring.

The main limitation is the limited availability of diverse real-world EVCS datasets containing both cyberattack and physical-fault events. The future study will focus on validation using larger real-world datasets, explainable AI, and real-time edge deployment. Overall, the proposed framework demonstrates strong potential for secure and reliable monitoring of IoT-enabled EV charging infrastructure.

## Data Availability

The original contributions presented in the study are included in the article/supplementary material, further inquiries can be directed to the corresponding author.
